# Fully automated deep learning based auto-contouring of liver segments and spleen on contrast-enhanced CT images

**DOI:** 10.1038/s41598-024-53997-y

**Published:** 2024-02-26

**Authors:** Aashish C. Gupta, Guillaume Cazoulat, Mais Al Taie, Sireesha Yedururi, Bastien Rigaud, Austin Castelo, John Wood, Cenji Yu, Caleb O’Connor, Usama Salem, Jessica Albuquerque Marques Silva, Aaron Kyle Jones, Molly McCulloch, Bruno C. Odisio, Eugene J. Koay, Kristy K. Brock

**Affiliations:** 1 Department of Imaging Physics, The University of Texas MD Anderson Cancer Center, Houston, TX USA; 2 The University of Texas MD Anderson Cancer Center UTHealth Graduate School of Biomedical Sciences, Houston, TX USA; 3 Abdominal Imaging Department, The University of Texas MD Anderson Cancer Center, Houston, TX USA; 4 Department of Radiation Physics, The University of Texas MD Anderson Cancer Center, Houston, TX USA; 5 Department of Interventional Radiology, The University of Texas MD Anderson Cancer Center, Houston, TX USA; 6 Department of Gastrointestinal Radiation Oncology, The University of Texas MD Anderson Cancer Center, Houston, TX USA

**Keywords:** Translational research, Computational science

## Abstract

Manual delineation of liver segments on computed tomography (CT) images for primary/secondary liver cancer (LC) patients is time-intensive and prone to inter/intra-observer variability. Therefore, we developed a deep-learning-based model to auto-contour liver segments and spleen on contrast-enhanced CT (CECT) images. We trained two models using 3d patch-based attention U-Net (${{\text{M}}}_{{\text{paU}}-{\text{Net}}})$ and 3d full resolution of nnU-Net (${{\text{M}}}_{{\text{nnU}}-{\text{Net}}})$ to determine the best architecture (${\text{BA}})$. BA was used with vessels (${{\text{M}}}_{{\text{Vess}}})$ and spleen (${{\text{M}}}_{{\text{seg}}+{\text{spleen}}})$ to assess the impact on segment contouring. Models were trained, validated, and tested on 160 (${{\text{C}}}_{{\text{RTTrain}}}$), 40 (${{\text{C}}}_{{\text{RTVal}}}$), 33 (${{\text{C}}}_{{\text{LS}}}$), 25 (C_CH_) and 20 (C_PVE_) CECT of LC patients. ${{\text{M}}}_{{\text{nnU}}-{\text{Net}}}$ outperformed ${{\text{M}}}_{{\text{paU}}-{\text{Net}}}$ across all segments with median differences in Dice similarity coefficients (DSC) ranging 0.03–0.05 (p < 0.05). ${{\text{M}}}_{{\text{seg}}+{\text{spleen}}}$, and ${{\text{M}}}_{{\text{nnU}}-{\text{Net}}}$ were not statistically different (p > 0.05), however, both were slightly better than ${{\text{M}}}_{{\text{Vess}}}$ by DSC up to 0.02. The final model, ${{\text{M}}}_{{\text{seg}}+{\text{spleen}}}$, showed a mean DSC of 0.89, 0.82, 0.88, 0.87, 0.96, and 0.95 for segments 1, 2, 3, 4, 5–8, and spleen, respectively on entire test sets. Qualitatively, more than 85% of cases showed a Likert score $\ge$ 3 on test sets. Our final model provides clinically acceptable contours of liver segments and spleen which are usable in treatment planning.

## Introduction

Liver cancer is the third most common cause of the cancer-related deaths globally and it resulted in roughly 700,000 deaths in 2020^1^. Surgery (resection or lobectomy) is considered the main line of treatment especially in colorectal liver metastases^2^ in which segment(s) or entire lobe is removed depending upon the extent of tumor^3,4^. However, the ability to perform liver surgery is largely dependent upon accurate localization of tumor with respect to segments and the volumetric measurement of liver segments as it allows clinician to ensure that the patient would have minimum remnant functional liver volume after the surgery (e.g. 20% in normal liver)^4^. To quantify the functional liver volume, radiologists/technologists perform manual contouring of segments on the contrast-enhanced CT (CECT) images following the architecture of vessels, ligament and organs^5^. However, manual contouring is time intensive^6^ and prone to inter/intra-observer variabilities^7^ which can affect the volumetric measurement and subsequent clinical use. Therefore, automation of liver segment contouring is crucial to evaluate the eligibility of patient for liver surgery.

Several semi-automatic and automatic segmentation approaches exist but recent advancements in Deep Learning (DL) based models have outperformed other methods in terms of required time and segmentation accuracies across various organ sites^8^. Recent surveys have reported a plethora of architectures used in medical image segmentation out of which U-Net based architectures are widely used for organ segmentations^9,10^. In particular, 3D U-Net (the 3D extension of U-Net) is of great importance as it offers two major features (1) training with sparse volumetric data (2) input of 3D volume/patch in the training which allows the architecture to retain more features in contrast to 2D input^11^. Both of those features make 3D U-Net more applicable in 3D organ segmentation, and resultingly, several studies^12,13^ have reported reasonable accuracy and clinically translatable performance of organ segmentations with 3D U-Net. Currently, nnU-Net is one of the state-of-art segmentation framework which utilizes U-Net based architectures (combined or individual 2D and 3D U-Net) to train segmentation models^14^, and has shown excellent translatable clinical performance in abdominal segmentation^15^. In addition, nnU-Net is a self-configuring framework and automatically performs hyperparameter tuning and data augmentation which promises to result in higher segmentation accuracies^14^. However, the presence of 3D input patch also implies the inclusion of features from irrelevant regions which involve large number of trainable parameters resulting in excessive requirement of computational resources. To address such issues, Attention based gating has been implemented by Oktay et al. 2018 in the standard 2D U-Net, which uses attention coefficients to identify relevant image features and merge them just before the concatenation operation in the skip-connection phase^16^. Additionally, Attention U-Net showed consistent significant performance improvements when its performance was compared with 3D U-Net^16^. However, since 3D input patch would also preserve higher number of relevant features compared to 2D input, it is therefore reasonable to implement attention mechanism in the multiple skip connection of standard “3D U-Net” and test if it would improve the segmentation accuracies.

Additionally, with regard to model training for segment contouring in the patients with primary and metastatic liver disease, the architecture has to face liver specific anatomical challenges which could result in uncertainties in demarcation of liver segments. For example, the occlusion of vessels due to tumor could result in distortion of liver contours and liver segments. Both aforementioned issues could be addressed if we can implement localization of vessels during the training. Another important condition is enlargement of liver and spleen in cancer patients in which spleen is abutted with segment 2 and 3 which result in incorrect separation of segments with spleen. One possible approach to address such issues is training the model with both segments and spleen. Currently, a very few DL based liver segmentation studies exist that investigated the automated segmentation accuracy on CT images of patients with liver tumors. Tian et al. 2019 implemented global and local context U-Nets (GLC-UNet) which first segmented the whole liver and then localized vessel-based slice features are utilized to segment the Couinaud’s segments^17^. GLC-UNet achieved a mean segment DSC similarity coefficient (DSCs) of 0.92. Additionally, a recent study by Lee et al. 2022 developed two different models to separately contour the liver segments and spleen and achieved a median DSC score around 0.91 across the segments^18^.

In this study, our central goal is to develop a fully automated segmentation model that can achieve consistent, robust, expert observer-level accuracy in liver segment contouring to guide the liver surgery planning. To achieve this goal, we have established three main aims (1) to determine the best architecture for auto segmentation of liver segments by investigating the performance of 3D patch based attention U-Net (paU-Net) over the gold-standard framework of nnU-Net (2) to determine if addition of vessels and spleen during segmentation training could improve the liver segments segmentations (3) to perform quantitative and qualitative assessment of model across patients undergoing RT, general evaluation for liver surgery, portal vein embolization (PVE), and CT based liver pathologies used in various segmentation challenges.

## Materials and methods

### Overall framework

**Figure 1:**
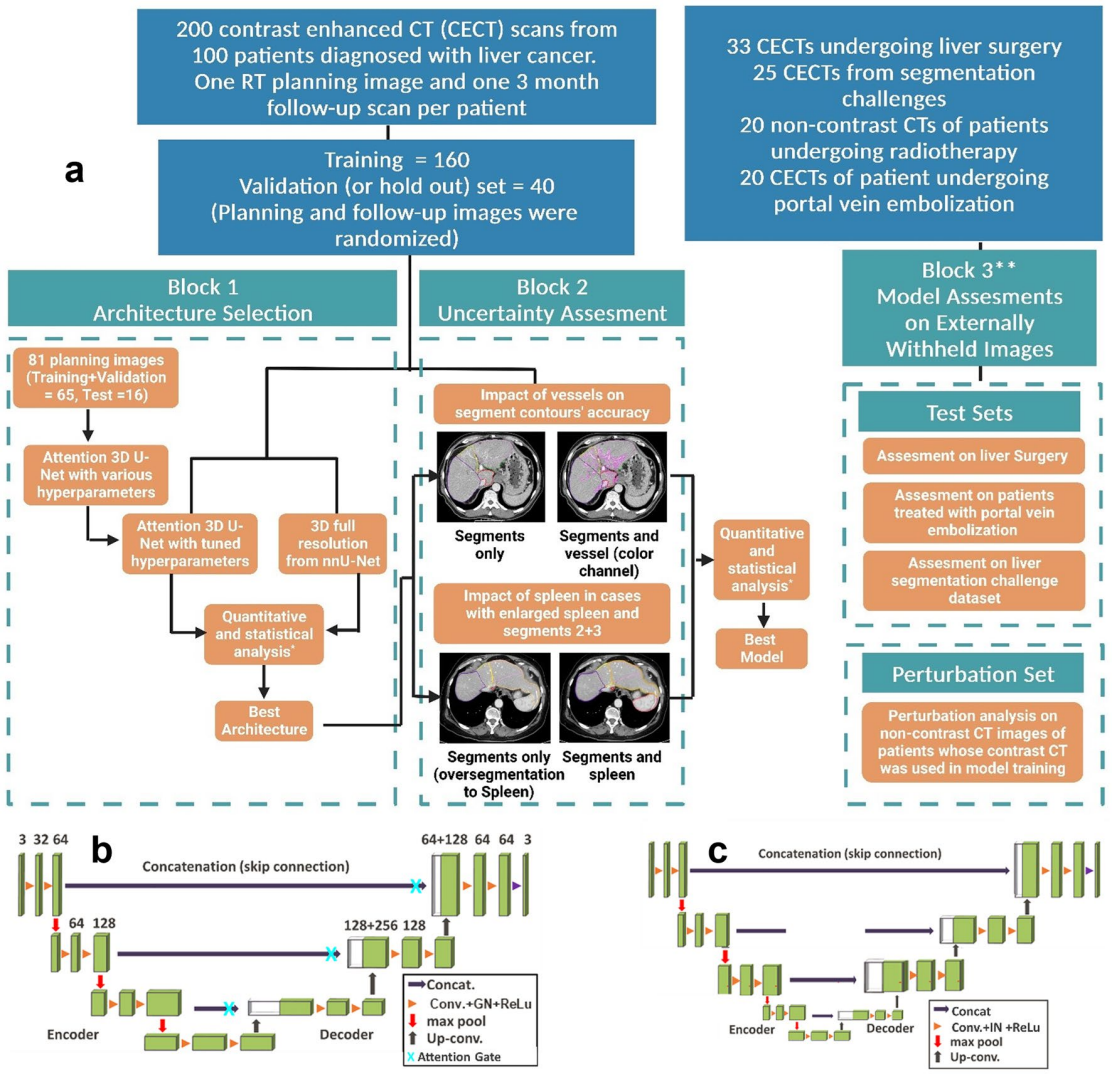
(**A**) Overall workflow of the study. (**B**) Architecture for 3D-patch based U-Net with attention mechanism (**C**) nnU-Net framework which automatically optimizes the architecture based on the type of datasets. *Quantitative analysis were performed by calculating Dice similarity coefficient, 95th percentile Hausdoff’s distance, and percent change in the volume of segments and spleen between AI predicted and ground-truth contours. Statistical analysis was performed using Wilcoxon signed rank test with Bonferroni correction. **All models were assessed on cohorts of Block 3 using both quantitative and qualitative analyses (Figures created using biorender.com).

Figure 1 shows the overall workflow of our study which involves three major blocks. Starting in the architecture selection block (block 1), we investigated the best architecture by comparing Attention 3D U-Net and 3D full resolution from nnU-Net. In the uncertainty improvement block (block 2), we investigated whether the addition of vessels and spleen during model training improves the segmentation results while using the best architecture identified from block 1. Lastly, in the Model Assessment (block 3), all the models were evaluated on surgery candidates’ CT scans, patients who received portal vein embolization, non-contrast CT images and on external CT datasets from various segmentation challenges^19–22^.

#### Datasets patient population

The study included two major data group, namely, an internal data group (IDG) and external data group (EDG). The IDG consisted of contrast enhanced CT (CECT) scans of patients diagnosed with primary and metastatic liver cancer at our institution. Within IDG, we have four cohorts. The radiotherapy cohort (${{\text{C}}}_{{\text{RT}}}$) consisted of 100 patients with a radiotherapy planning and 3-month follow-up CECT image. The surgery cohort (${{\text{C}}}_{{\text{LS}}}$) included 33 CT scans of patients that were being evaluated for liver surgery. The non-contrast cohort (${{\text{C}}}_{{\text{NC}}}$) included 20 non-contrast CT (non-CECT) scans of patient with contrast scans used in the training. The portal vein embolization cohort (${{\text{C}}}_{{\text{PVE}}}$) included 20 CT scans of patient undergoing portal vein embolization for the liver (PVE). All patients from internal data group were retrospectively enrolled in a Health Insurance Portability and Accountability Act-compliant institutional review board approved study (The University of Texas MD Anderson Cancer Center IRB PA18-0832) with a waiver of informed consent. Use of data was approved by the IRB and all experiments were performed in accordance with relevant guidelines and regulations.

The EDG consisted of challenge data (${{\text{C}}}_{{\text{CH}}}$) which included a total of 25 patients obtained from 3D-IRCADb-01 (${{\text{C}}}_{{\text{IRCAD}}-01}$), 3D-IRCADb-02 $({{\text{C}}}_{{\text{IRCAD}}-02}$), task 8 Medical Imaging Decathlon Challenge (${{\text{C}}}_{{\text{MID}}}$) and CHAOS (${{\text{C}}}_{{\text{CHAOS}}}$) datasets^19–21^. Table 1 shows the detailed technical information regarding the images and patients used in this study.

**Table 1:** Characteristics of patients used in this study.

Cohorts (Number of images)	Treatment type	Used for	Image	Cancer types** (Number of patients)	Median Voxel size (in x/y, mm)^┼^	Median Voxel size (in z, mm)^┼^
${{\text{C}}}_{{\text{RTTrain}}}$(N = 160)	Radiotherapy	Training	Contrast	HCC = 30 CC = 49 CRM = 12 mixed = 4	0.98 (0.66–1.17)	2.5 (0.63–5.0)
${{\text{C}}}_{{\text{RTVal}}}$(N = 40)	Radiotherapy	Validation	Contrast	HCC = 16 CC = 18 CRM = 2	0.98 (0.7–1.07)	2.5 (2.5–5.0)
${{\text{C}}}_{{\text{LS}}}$(N = 33)	Liver surgery evaluation	Test	Contrast	HCC = 2 CC = 5 CRM = 22	0.86 (0.51–0.98)	2.5 (2.5–5.0)
${{\text{C}}}_{{\text{PVE}}}$(N = 20)	Portal vein embolization	Test	Contrast	HCC = 0 CC = 3 CRM = 17	0.80 (0.70–0.98)	2.5 (1.0–2.5)
${{\text{C}}}_{{\text{CH}}}$(N = 25)	Liver segmentation challenge	Test	Contrast	NA	0.71 (0.60–0.96)	2 (1.0–5.0)
${{\text{C}}}_{{\text{CNC}}}$(N = 20)	Pre-contrast of ${{\text{C}}}_{{\text{RT}}}$	Perturbation study	Non-contrast	HCC = 6 CC = 10 CRM = 3 mixed = 1	0.98 (0.98–1.17)	2.5 (all*)

^┼^Median (min–max).

*All means all the cases showed same values.

**CRM colorectal or other metastasis, *CC* Cholangiocarcinoma, *HCC* Hepatocellular carcinoma, *mixed* more than one cancer types.

#### Manual and AI edited segmentations

Ground-truth segmentations of the patient datasets included liver segments 1, 2, 3, 4, 5–8 (combined), spleen, and vessels. Two major approaches were used to contour the liver segments. In first approach, an in-house nnU-Net model trained on the subset of ${{\text{C}}}_{{\text{RTTrain}}}$ was used to contour liver segments on ${{\text{C}}}_{{\text{RT}}}$ and ${{\text{C}}}_{{\text{LS}}}$. Afterwards, the model generated contours were edited or recontoured fully by a radiologist (MA) as per the need. In second approach, liver segments were manually contoured by the radiologist MA on ${{\text{C}}}_{{\text{PVE}}}$, and ${{\text{C}}}_{{\text{CH}}}$ without any assistance from AI models. Additionally, spleen contours on ${{\text{C}}}_{{\text{RT}}}$ and ${{\text{C}}}_{{\text{CH}}}$ were first created by a nnU-Net model trained on task 9 Medical Imaging Decathlon Dataset^20^ and were manually edited by a radiologist (MA) or students (SR and ACG). On ${{\text{C}}}_{{\text{PVE}}}$, ${{\text{C}}}_{{\text{LS}}}$, and ${{\text{C}}}_{{\text{NC}}}$ the spleen was manually contoured by ACG without using any AI segmentations. Lastly, the reader is referred to Sect. “Uncertainty improvement-impact of vessels and spleen” for mechanism behind vessels contours.

#### Architecture selection

We have investigated two variants of 3D U-Net in this study. First, we developed a 3D patch-based U-Net with attention mechanism based on the standard 3D U-Net^11^ and attention gate^16^. As shown in Fig. 1A and B, in the analysis path, a patch size of 256 × 256 × 24 was input to the network. The network consisted of 4 layers with 2 blocks in each layer. A convolution of 3 × 3 × 3 is performed at each block with group normalization and Leaky ReLU followed by a 2 × 2 × 2 max pooling before transitioning to the next layer. In the decoder, blocks within each layer undergo up-sampling through convolution of 3 × 3 × 3. A skip-layer with concatenation is implemented which feeds the feature map from corresponding block in encoder to attention gate. The attention gate suppresses the irrelevant features and noise as per the standard methodology^16^. The gated feature is then concatenated to the transposed block in the analysis. A final 1 × 1 × 1 convolution is performed in the last layer of the decoder path to produce the image with selected number of classes. Categorical cross entropy is used as the loss function for validation. To identify the best hyperparameters, we performed multiple trainings (epoch = 1000) using stable and cyclic learning rates (rate = 0.0001) for number of blocks = 2 and 3 and number of filters = 16, 32, 48, 64. As a result, 16 models were trained.

Second, we investigated the 3D full-resolution configuration of nnU-Net which is also a patch-based 3D U-Net. nnU-Net automatically generates the segmentation pipeline specific to the dataset through its three major domains: fixed, rule-based, and empirical parameters, which handles all the preprocessing, training and postprocessing for the datasets^14^. Unlike our in-house architecture, the nnU-Net automatically selects the hyperparameter that is suitable for a dataset. Figure 1C shows an example of nnU-Net architecture which was used to train the model in section. A patch size of 192 × 192 × 48 with a batch size of 2 is input to the architecture with 5 layers, 2 blocks, and 32 filters. In the encoder, there is a convolution of 3 × 3x × 3 followed by Intensity Normalization (IN) and a 2 × 2 × 2 max pooling. In the decoder, blocks undergo up-sampling using the same mechanism as described for the 3D U-Net. Data augmentation was performed automatically as described in the nnU-Net guidelines^14^. Combined DSC and cross-entropy are used as the loss function.

To identify the best architecture, we trained two models, one based on the patch U-Net (M_paU-Net_) and one based on the nnU-Net (M_nnU-Net_) to predict the segmentation of segments 1, 2, 3, 4, and 5–8. Models were trained for five-fold cross validation using ensemble approach in both architectures. In M_paU-Net_, majority vote and STAPLE algorithm from Simple ITK v2.2.1 was implemented to select the best result from five folds. In M_nnU-Net_, the default configuration of nnU-Net (average ensembling) was used^14^. Quantitative and statistical analysis were performed (as per Sect. “Data analysis”) to select the best architecture model, M_Best-Architecture_.

#### Uncertainty improvement-impact of vessels and spleen

We investigated if the uncertainties in the definition of liver segment boundaries can be improved by incorporating two additional features in the training.

First, we trained a model (${{\text{M}}}_{{\text{vess}}}$) using ${{\text{M}}}_{{\text{Best}}-{\text{Architecture}}}$ (from Block 1) to investigate if the incorporation of vessels during the training would improve the segmentation of the liver segments. We began by generating liver vessels using the liver vessel generation algorithms^23^ available in a commercial treatment planning system (RayStation v12.0.110.72, RaySearch Laboratories, Stockholm, Sweden) on ${{\text{C}}}_{{\text{RT}}}$ (N = 200). The binary label map of vessels was added as an extra input channel using modality function in nnU-Net, and the model was trained to predict the contours of liver segments. Second, we trained a model (${{\text{M}}}_{{\text{seg}}+{\text{spleen}}}$) using ${{\text{M}}}_{{\text{Best}}-{\text{Architecture}}}$ (from Block 1) to determine if the addition of spleen contours during the training would result in improved segmentation of liver segments, especially segments 2 and 3. The training was optimized to predict the contours of the liver segments (with segments 5–8 combined) and spleen.

Last, we individually compared the performance of the models ${{\text{M}}}_{{\text{ves}}}$ and ${{\text{M}}}_{{\text{seg}}+{\text{spleen}}}$ with our best architecture model ${{\text{M}}}_{{\text{Best}}-{\text{Architecture}}}$ to determine if individual features improved the segmentation performance. Additionally, we compared ${{\text{M}}}_{{\text{vess}}}$ and ${{\text{M}}}_{{\text{seg}}+{\text{spleen}}}$ models to determine if one features would result in greater impact on segmentation. To select a single best model (${{\text{M}}}_{{\text{Best}}-{\text{Model}}})$, all the model comparisons were performed on the external validation set ${{\text{C}}}_{{\text{RT}}}{\text{val}}$ using quantitative and qualitative assessment described in Sect. “Quantitative analysis” and “Qualitative analysis”. After the optimal model was selected, all models were evaluated on all test sets to determine if the optimal model ranking was held in the test environment.

### Training, validation and test set for model creation

Our framework includes training, validation, and test sets. As shown in Fig. 1A, C_RT_ was used for training and validation, and C_LS_, C_CH_, C_PVE_ were used for test set. C_RT_ datasets were split into training (N = 160), and validation (N = 40) by randomizing planning and 3 month follow up images of patients. The optimization of models during training was performed using cross entropy and dice as a loss function (see Sect. “Manual and AI edited segmentations” for more details). Hyperparameters were tuned manually and automatically according to architecture as described in Sect. “Manual and AI edited segmentations”. All models in our study were trained for 1000 epochs and with five-fold cross-validation. All models were evaluated on both validation (${{\text{C}}}_{{\text{RTVal}}}$) and test sets (${{\text{C}}}_{{\text{LS}}}, {{\text{C}}}_{{\text{CH}}}, {{\text{C}}}_{{\text{PVE}}}$). While the main purpose of validation set was to select the best model, the assessment of models on test set was used to further establish the discrimination among the model performance.

Table 2 shows the labels used in the study and data separation for training, validation, and test across the models.

**Table 2:** Number of CT scans allotted for training, validation, and test sets across different models.

Model	Training	Validation (${{\text{C}}}_{{\text{RT}}}{\text{val}}$)	Global test sets (C_LS_, C_PVE_, C_CH_)	Labels
M_paU-Net_	160	40	33 + 20 + 25	Segments 1, 2, 3, 4, 5-8
M_nnU-Net_	160	40	33 + 20 + 25	Segments 1, 2, 3, 4, 5-8
M_vess_	160	40	33 + 20 + 25	Segments 1, 2, 3, 4, 5-8, vessels as color channel
M_seg+spleen_	160	40	33 + 20 + 25	Segments 1, 2, 3, 4, 5-8 Spleen

### Assessment of the models on patients withheld from training/validation

#### Assessment of the final model on the liver surgery patients

To assess the accuracy of the models in clinical practice, we retrospectively obtained 33 CT scans of patients for whom the segment volume was assessed to determine the eligibility of the patient for liver surgery. AI predicted contours from each model were quantitatively and qualitatively evaluated as per Sect. “Qualitative analysis”.

#### Assessment of the models on challenge datasets

This test set was developed by randomly selecting 25 CT images from each cohort ${{\text{C}}}_{{\text{IRCAD}}-01}, {{\text{C}}}_{{\text{IRCAD}}-02}, {{\text{C}}}_{{\text{MID}}},$ and ${{\text{C}}}_{{\text{CHAOS}}}$. A radiologist (MA) contoured the liver segments and spleen on each CT. The liver segment and spleen contours generated by all of the models were qualitatively and quantitatively compared with the ground-truth contours.

#### Assessment of the models on post-portal vein embolization images

This test set was developed by obtaining 20 patients who received Portal vein embolization at our institution. This analysis's main purpose was to quantify the model's performance in presence of liver hypertrophy and metallic artifacts. All images included some form of metallic artifacts due to embolization coil. AI predicted contours from all models were assessed against the ground-truth using both quantitative and qualitative analysis.

#### Perturbation analysis of the model using non-contrast images

Here, we investigated the adaptability of our models on the perturbed images of patients using non-contrast images which is one of the clinical scenarios. We randomly selected CECT images of 20 patients used in training and then obtained their corresponding pre-contrast CT (i.e., non-CECT) images from the same four-phase liver CT protocol examination. To generate the ground-truth contours of liver segments, we first contoured the whole liver on the both CECT and non-CECT using our deep-learning based model^24^, and then performed whole liver based biomechanical deformable image registration using an algorithm previously validated^25,26^. We used models ${{\text{M}}}_{{\text{Best}}-{\text{Architecture}}}$ and ${{\text{M}}}_{{\text{seg}}+{\text{spleen}}}$ to predict the liver segments and spleen.${{\text{M}}}_{{\text{vess}}}$ was not assessed because non-contrast images lack the vessels in the image. Further, no qualitative analysis was performed due to absence of vessel information on the image. In addition to quantitative metrics mentioned in Sect. “Qualitative analysis”, mean distance to agreement (MDA) was also evaluated to further quantify the adaptability of our model when presented with perturbation.

### Data analysis

#### Quantitative analysis

The performance of the model was evaluated on all validation (N = 40), entire test (N = 78 total) and perturbation sets (N = 20) using Sorenson-DSC similarity coefficients (DSC), average Hausdorff Distance (HD_A_), 95th Percentile Hausdorff Distance (HD_95_), Percent Difference in the Volume (PDV).

For further comparison, we calculated the individual DSC differences (${{\text{DSC}}}_{{{\text{M}}}_{1}-{{\text{M}}}_{2}}$) between the corresponding cases of models of interests using Eq. (1a) and binned the results in [0.025, 0.05), [0.05, 0.1), and [0.1, 1) under respective models based on the sign (Eq. (1b)). Lastly, the ratio of the frequency of cases within each bin from two models of interests was used to evaluate the models (Eq. (2)).

1a $${{\text{DSC}}}_{{{\text{M}}}_{1}-{{\text{M}}}_{2}}= {{\text{DSC}}}_{{{\text{M}}}_{1}}-{{\text{DSC}}}_{{{\text{M}}}_{2}}$$

1b $${{\text{DSC}}}_{{{\text{M}}}_{1}-{{\text{M}}}_{2}}\upepsilon \left\{\begin{array}{c}{{{\text{N}}}_{{\text{M}}}}_{1}, {{\text{DSC}}}_{{{\text{M}}}_{1}-{{\text{M}}}_{2}}\ge 0.025\\ {{{\text{N}}}_{{\text{M}}}}_{2},{\mathrm{ DSC}}_{{{\text{M}}}_{1}-{{\text{M}}}_{2}} \le -0.025\end{array}\right.$$

2 $${{\text{f}}}_{{{\text{M}}}_{1}:{{\text{M}}}_{2}}=\frac{{{\text{N}}}_{{\text{M}}1}}{{{\text{N}}}_{{\text{M}}2}}$$

where ${{\text{M}}}_{1}$ and ${{\text{M}}}_{2}$ are two models of interests and could be any models from {${{\text{M}}}_{{\text{paU}}-{\text{Net}}}, {{\text{M}}}_{{\text{nnU}}-{\text{Net}}}, {{\text{M}}}_{{\text{vess}}}, {{\text{M}}}_{{\text{seg}}+{\text{spleen}}}$}. ${{{\text{N}}}_{{\text{M}}}}_{1}$ and ${{{\text{N}}}_{{\text{M}}}}_{2}$ are number of cases from each model meeting the criteria in Eq. (2). All parameters discussed above were assessed for segmentations corresponding to the models in Table 2.

#### Qualitative analysis

Unipolar Likert scale survey on the scale of 1–5 was performed by radiologists to evaluate the contours from various datasets. To avoid the inherent biasness in observer, the assessments were performed by two radiologist who did not participate in delineating any contours in our study. A radiologist (SY) evaluated the contours of all models on ${{\text{C}}}_{{\text{LS}}}$ and ${{\text{C}}}_{{\text{CH}}}$. Another radiologist (US) evaluated the contours of all models on ${{\text{C}}}_{{\text{RTVal}}}$ and ${{\text{C}}}_{{\text{PVE}}}$. Likert scoring criteria with the definition of rating is shown in the Table 3 below.

**Table 3﻿:** Scoring criteria used by radiologists to evaluate the contours for qualitative analysis.

	Likert scale	Criteria
5	Strongly agree	Minor edits which are not clinically important, or no edits are required. Can use the contours in the clinic without any edits
4	Agree	Minor edits (peripheral portal veinous branches) are required, and the time required to recontour is minimal
3	Neither agree nor disagree	Major edits (Major vessels (hepatic veins, right/left/main portal, and segmental portal vein branches) boundaries and anatomical boundaries (intersegmental fissure and gall bladder fossa) need to be corrected) but time required to recontour is minimal
2	Disagree	Major edits are required, and the time required to edit the contour is extraordinarily long
1	Strongly disagree	Segmentations are unusable

#### Intra- and inter-observer analysis

We selected 10 images that were used in our model training. Radiologist MA contoured the segments twice in the gap of two weeks and relative inter-observer variability in DSC was estimated. Additionally, another radiologist, JAMS, contoured the liver segments on the same patients, and relative interobserver variability in DSC were calculated with respect to the contours of MA.

#### Statistical analysis

Wilcoxon signed-rank test was performed to determine if the models were statistically different (p < 0.05). For comparison involving more than 2 models, Bonferroni correction was performed to adjust the p-values.

## Results

### Selection of best architecture

The best tuned hyperparameters for paU-Net were obtained for the model with 3 blocks and 64 filters. This model showed highest validation DSC of 0.75 and a low difference between training and validation DSC of 0.14 among all paU-Net models.

In paU-Net’s ensembling method comparison, the majority vote and STAPLE based contours showed overall similar mean DSCs of 0.86 and 0.87, respectively. However, when we compared minimum DSC of segments altogether, STAPLE showed improvement of 0.052 or 5.2% on average (see Table S1). Additionally, our visual assessment revealed that the majority vote contours had increased zero voxels at the boundaries of segments compared to STAPLE results (see Fig. S1). Therefore, we selected STAPLE based prediction as our final ensembling method for ${{\text{M}}}_{{\text{paU}}-{\text{Net}}}$.

Table 4 shows the volumetric and overlap metric comparison between the results of ${{\text{M}}}_{{\text{paU}}-{\text{Net}}}$ and ${{\text{M}}}_{{\text{nnU}}-{\text{Net}}}$. ${{\text{M}}}_{{\text{paU}}-{\text{Net}}}$ and ${{\text{M}}}_{{\text{nnU}}-{\text{Net}}}$ showed overall mean (average of median) DSC of 0.87 (0.87) and 0.89 (0.92), respectively, when assessed across all segments. The individual mean DSC values of ${{\text{M}}}_{{\text{nnU}}-{\text{Net}}}$ for segments 1, 2, 3, and 4 were greater than that of ${{\text{M}}}_{{\text{paU}}-{\text{Net}}}$ by 0.03, 0.04, 0.02, and 0.05, respectively. The ratio of number of cases meeting binned differences (Eq. (3)) i.e., ${{\text{f}}}_{{{\text{M}}}_{{\text{nnU}}-{\text{Net}}}:{{\text{M}}}_{{\text{paU}}-{\text{Net}}}}$ was > 3 for segments 2, 3, and 5–8 and were > 10 for segments 2 and 4 (see Table S2 for details). Additionally, ${{\text{M}}}_{{\text{nnU}}-{\text{Net}}}$ demonstrated lower mean and median HD_95_ values than ${{\text{M}}}_{{\text{paU}}-{\text{Net}}}$ for each segment. The difference in mean and median HD_95_ between ${{\text{M}}}_{{\text{paU}}-{\text{Net}}}$ and ${{\text{M}}}_{{\text{nnU}}-{\text{Net}}}$ were within 1 mm for all segments except segment 4 where the differences were 16.3 mm (mean) and 2.7 mm (median), with ${{\text{M}}}_{{\text{nnU}}-{\text{Net}}}$ having superior performance. PDV comparison showed that differences in mean and median were mostly within $\pm$ 1.5% with few exceptions; segment 1 showed differences of − 5.3% and − 3.2% for mean and median, respectively, with ${{\text{M}}}_{{\text{nnU}}-{\text{Net}}}$ having superior performance, segment 2 showed − 5.8% (mean) and segment 4–3.9%(mean), with ${{\text{M}}}_{{\text{nnU}}-{\text{Net}}}$ having superior performance. Statistically, Wilcoxon signed-rank showed that performance difference of the models were significant for DSC values of all segments with ${{\text{M}}}_{{\text{nnU}}-{\text{Net}}}$ having superior performance. Further, except segments 2 and 5–8 in ${{\text{HD}}}_{{\text{A}}}$ and ${{\text{HD}}}_{95}$, all other metrics/segments showed statistical significance in the comparison. Lastly, as per the qualitative assessment (Table 5), 99% of cases from ${{\text{M}}}_{{\text{nnU}}-{\text{Net}}}$ received an overall score $\ge$ 3 whereas 88% of cases from ${{\text{M}}}_{{\text{paU}}-{\text{Net}}}$ received an overall score of $\ge$ 3. Considering the better agreement with ${{\text{M}}}_{{\text{nnU}}-{\text{Net}}}$ qualitatively and quantitatively, we selected nnU-Net as the best architecture, i.e. ${{\text{M}}}_{{\text{Best}}-{\text{Architecture}}}={{\text{M}}}_{{\text{nnU}}-{\text{Net}}}$. Hereafter, ${{\text{M}}}_{{\text{nnU}}-{\text{Net}}}$ is also used to represent the best architecture which is nnU-Net model trained with segments only.

**Table 4:** Comparison of descriptive statistics from paU-Net and nnU-Net.

	Seg 1 N = 39^2^		Seg 2 N = 40		Seg 3 N = 40		Seg 4 N = 40		Seg 5–8 N = 40	
	paU-Net^1^	nnU- Net^1^	paU-Net^1^	nnU- Net^1^	paU-Net^1^	nnU- Net^1^	paU-Net^1^	nnU- Net^1^	paU-Net^1^	nnU- Net^1^
DSC	0.90	0.93	0.82	0.86	0.89	0.91	0.86	0.91	0.97	0.97
	0.88	0.90	0.81	0.83	0.87	0.89	0.83	0.88	0.96	0.97
	0.07	0.07	0.08	0.09	0.09	0.10	0.11	0.08	0.03	0.03
	0.96	0.98	0.92	0.94	0.94	0.96	0.95	0.97	0.99	0.99
	0.71	0.70	0.56	0.51	0.44	0.36	0.46	0.56	0.88	0.86
	**		*		**		**		**	
${{\text{HD}}}_{95}^{3}$	4	3.1	8	8.0	8	6.6	9	6.5	6	4.5
	5	4.6	11	9.5	9	8.0	25	8.3	10	7.6
	5	6.1	8	6.0	5	5.5	34	6.7	11	8.8
	31	37.1	34	33.4	26	28.9	118	36.7	46	47.8
	1	0.8	4	3.1	4	2.5	4	2.1	1	0.7
	*		*ns*		*ns*		**		*ns*	
${{\text{HD}}}_{{\text{A}}}^{3}$	0.20	0.14	0.60	0.48	0.38	0.31	0.49	0.24	0.09	0.06
	0.35	0.30	0.79	0.69	0.57	0.49	1.18	0.47	0.22	0.18
	0.50	0.61	0.61	0.62	0.81	0.87	1.78	0.55	0.37	0.29
	2.99	3.85	2.55	2.92	4.56	5.37	9.43	2.71	1.85	1.54
	0.06	0.02	0.19	0.11	0.14	0.08	0.11	0.04	0.01	0.01
	**		*ns*		*		**		*ns*	
PDV	10	7	16	11	6	5	7	7	3	2
	14	8	22	21	9	8	12	8	3	4
	15	10	22	35	8	8	13	7	3	4
	80	45	123	173	39	27	54	30	19	22
	0	0	1	0	0	0	0	0	0	0
	*		*ns*		*ns*		**		*ns*	

^1^Data in each cell is organized as row 1 = Median, row 2 = Mean, row 3 = Standard deviation, row 4 = Max, row 5 = Min row row 6 = significance level, P $\le 0.05$ = *, P $\le 0.01$ = **, P $>$ 0.05 = ns.

^2^One of the patients was removed because paU-Net failed to predict segment 1.

^3^${{\text{HD}}}_{{\text{A}}}$ and ${{\text{HD}}}_{95}$ average and 95% Hausdorff distance (mm); PDV = percent difference in volume.

### Impact of vessels and spleen on segment contouring/selection of best model

Tables 5 and 6 shows the comparison of models ${{\text{M}}}_{{\text{nnU}}-{\text{Net}}}$, ${{\text{M}}}_{{\text{vess}}}$, and ${{\text{M}}}_{{\text{seg}}+{\text{spleen}}}$ using quantitative and qualitative approach described in Sects. “Quantitative analysis” and “Qualitative analysis”, respectively. For ${{\text{M}}}_{{\text{vess}}}$ vs.${{\text{M}}}_{{\text{nnU}}-{\text{Net}}}$, ${{\text{M}}}_{{\text{vess}}}$ showed DSC values of 0.89 (mean) and 0.91 (average of median), which are similar to mean DSC of 0.89 and average of median DSC of 0.92 of ${{\text{M}}}_{{\text{nnU}}-{\text{Net}}}$. Individual DSC difference (${{\text{M}}}_{{\text{vess}}}- {{\text{M}}}_{{\text{nnU}}-{\text{Net}}}$) were within -0.01 (mean) for segments 2, 3, 4, 5–8 and − 0.02 (median) for segments 2 and 4. All other segments had mean and median DSC difference of 0. ${{\text{f}}}_{{\text{M}}_{{\text{vess}}}:{{\text{M}}}_{{\text{nnU-Net}}}}$ was $\le 1:3 (0.33)$ (see Table S3 for details) for all except segment 5–8 where the ratio was 1:1. With regard to ${{\text{HD}}}_{{\text{95}}}$, the difference in mean and median values (${{\text{M}}}_{{\text{vess}}}- {{\text{M}}}_{{\text{nnU}}-{\text{Net}}}$) were within $\pm 1mm$ for all cases except mean values of segments 3, 4, and 5–8 where the differences were 2.6 mm,1.50 mm, and 1.67 mm, respectively. Additionally, the overall differences in the mean and median PDV values were within $\pm 2.5\%.$ Most of the differences were $>0$, indicating a reduction in performance for ${{\text{M}}}_{{\text{vess}}}$. Qualitatively, the difference between the cases of ${{\text{M}}}_{{\text{vess}}}$ and ${{\text{M}}}_{{\text{nnU}}-{\text{Net}}}$ receiving score $\ge$ 3 is within 1% in all segments except segments 2 and 3 where ${{\text{M}}}_{{\text{nnU}}-{\text{Net}}}$ leads by 3% and 5% respectively. Overall, the metrics of ${{\text{M}}}_{{\text{vess}}}$ were equivalent or slightly worse than of that of ${{\text{M}}}_{{\text{nnU}}-{\text{Net}}}$.

**Table 5:** Comparison of descriptive statistics from models trained with segments, segments with vessel (color channel) and segments with spleen on validation set (C_RTVal_).

	Seg 1,			Seg 2,			Seg 3,			Seg 4,			Seg 5–8,			Spleen, N = 40^1^
	N = 40			N = 40^1^			N = 40^1^			N = 40^1^			N = 40^1^			
	(IntraMD^2a^ = 0.88)			(IntraMD^2a^ = 0.88)			(IntraMD^2a^ = 0.94)			(IntraMD^2a^ = 0.92)			(IntraMD^2a^ = 0.99)			
	(InterMD^2b^ = 0.82)			(InterMD^2b^ = 0.85)			(InterMD^2b^ = 0.91)			(InterMD^2b^ = 0.88)			(InterMD^2b^ = 0.96)			
	M_nnUnet_^1^	M_seg+spleen_^1^	M_vess_^1^	M_nnUnet_^1^	M_seg+spleen_^1^	M_vess_^1^	M_nnUnet_^1^	M_seg+spleen_^1^	M_vess_^1^	M_nnUnet_^1^	M_seg+spleen_^1^	M_vess_^1^	M_nnUnet_^1^	M_seg+spleen_^1^	M_vess_^1^	M_seg+spleen_^1^
Dice	0.93	0.93	0.93	0.86	0.86	0.84	0.91	0.91	0.91	0.91	0.90	0.89	0.97	0.97	0.97	0.99
	0.90, 0.07	0.90, 0.06	0.90, 0.07	0.83, 0.09	0.83, 0.10	0.82, 0.10	0.89, 0.10	0.89, 0.10	0.88, 0.12	0.88, 0.08	0.88, 0.08	0.87, 0.09	0.97, 0.03	0.97, 0.02	0.96, 0.03	0.99, 0.01
	0.98, 0.70	0.98, 0.75	0.97, 0.73	0.94, 0.51	0.94, 0.48	0.93, 0.47	0.96, 0.36	0.96, 0.36	0.96, 0.21	0.97, 0.56	0.97, 0.55	0.97, 0.57	0.99, 0.86	0.99, 0.91	0.99, 0.84	1.00, 0.96
${\text{HD}}_{95}^{3}$	3.1	3.1	3	8.0	7.5	8	6.6	6.5	7	6.5	6.2	7	4.5	4.4	6	0.9
	4.6, 6.1	4.6, 5.7	5, 6	9.5, 6.0	8.9, 5.1	9, 5	8.0, 5.5	8.4, 6.7	11, 13	8.3, 6.7	8.4, 6.7	10, 8	7.6, 8.8	7.0, 7.5	9, 12	0.9, 0.7
	37.1, 0.8	34.4, 0.8	34, 1	33.4, 3.1	25.8, 2.7	30, 3	28.9, 2.5	32.3, 2.5	81, 2	36.7, 2.1	36.5, 1.5	41, 2	47.0, 0.7	38.2, 0.7	54, 1	2.7, 0.0
Avg. ${\text{HD}}_{{\text{A}}}^{3}$	0.14	0.13	0.15	0.48	0.45	0.57	0.31	0.28	0.32	0.24	0.24	0.33	0.06	0.06	0.08	0.01
	0.30, 0.61	0.28, 0.50	0.29, 0.50	0.69, 0.62	0.66, 0.60	0.70, 0.60	0.49, 0.87	0.52, 0.97	0.64, 1.33	0.47, 0.55	0.47, 0.52	0.64, 0.90	0.18, 0.29	0.13, 0.19	0.27, 0.61	0.02, 0.01
	3.85, 0.02	3.14, 0.02	3.16, 0.03	2.92, 0.11	3.16, 0.15	3.02, 0.16	5.37, 0.08	5.54, 0.08	7.97, 0.09	2.71, 0.04	2.33, 0.04	4.57, 0.05	1.54, 0.01	1.03, 0.01	3.38, 0.01	0.06, 0.00
PDV	7	5	6	11	10	10	5	5	5	7	6	8	2	2	3	0
	8, 10	8, 10	9, 12	21, 35	21, 36	23, 40	8, 8	8, 8	9, 9	8, 7	9, 7	11, 10	4, 4	3, 3	4, 4	1, 1
	45, 0	45, 0	63, 0	173, 0	177, 0	218, 0	27, 0	29, 0	30, 0	30, 0	25, 1	52, 1	22, 0	12, 0	18, 0	4, 0

^1^Data in each cell is organized as row 1 = Median, row 2 = Mean, Standard deviation, row 3 = Max, Min; ^1^M_nnUnet_ = model trained with segments only, M_seg+spleen_ = Model trained with Segments and Spleen as labels, M_vess_ = Model trained with segments as label and vessel as color channel.

^2a^IntraMD = Intra-observer mean dice.

^2b^InterMD = Inter-observer mean dice.

^3^HD = Hausdorff distance (95 = 95th percentile and A = Average in mm); Wilcoxon signed rank test with Bonferroni adjustment showed p > 0.05 in M_nnU-Net_ vs. M_seg+spleen_ for all. In M_seg+spleen_ vs. M_vess_, segment 3 showed p < 0.05 in DSC and HD_A_. In M_nnU-Net_ vs. M_vess_, segment 3 showed p < 0.05 in HD_95_ and segment 4 showed p < 0.05 in PDV and HD_95_.

**Table 6:** Likert scale assessment performed by independent radiologists to asses the usability of contours in the clinic.

Challenge cohort (C_CH_)					Validation cohort (C_RTval_)				Surgery cohort (C_LS_)				Portal vein embolization cohort (C_PVE_)			
Scores	Segment and Vessels with nnU-Net (M_vess_), N = 25^*1*^	Segment Only with nnU-Net (M_nnU-Net_), N = 25^*1*^	Segment only with paU-Net (M_paU-Net_), N = 25^*1*^	Segments and Spleen with nnU-Net (M_seg+spleen_), N = 25^*1*^	Segment and Vessels with nnU-Net (Mvess), N = 40^*1*^	Segment Only with nnU-Net (M_nnU-Net_), N = 40^*1*^	Segment only with paU-Net (M_paU-Net_), N = 40^*1*^	Segments and Spleen with nnU-Net (M_seg + Spleen_), N = 40^*1*^	Segment and Vessels with nnU-Net (M_Vess_), N = 31^*1*^	Segment Only with nnU-Net (M_nnU-Net_), N = 33^*1*^	Segment only with paU-Net (M_paU-Net_), N = 33^*1*^	Segments and Spleen with nnU-Net (M_seg + Spleen_), N = 33^*1*^	Segment and Vessels with nnU-Net (M_Vess_), N = 20^*1*^	Segment Only with nnU-Net (M_nnU-Net_), N = 20^*1*^	Segment only with paU-Net (M_paU-Net_), N = 20^*1*^	Segments and Spleen with nnU-Net (M_seg + Spleen_), N = 20^*1*^
Seg 1																
1	0	0	0	0	1 (2.5%)	1 (2.5%)	1 (2.6%)	1 (2.5%)	1 (3.2%)	1 (3.0%)	4 (12%)	1 (3.0%)	0	0	0	0
2	0 (0%)	0 (0%)	4 (16%)	0 (0%)	0	0	0	0	0	0	0	0	0 (0%)	0 (0%)	1 (5.0%)	0 (0%)
3	4 (16%)	4 (16%)	4 (16%)	4 (16%)	1 (2.5%)	1 (2.5%)	1 (2.6%)	1 (2.5%)	7 (23%)	8 (24%)	6 (18%)	7 (21%)	1 (5.0%)	1 (5.0%)	0 (0%)	1 (5.0%)
4	13 (52%)	13 (52%)	14 (56%)	13 (52%)	11 (28%)	11 (28%)	11 (28%)	11 (28%)	14 (45%)	14 (42%)	17 (52%)	15 (45%)	1 (5.0%)	2 (10%)	5 (25%)	1 (5.0%)
5	8 (32%)	8 (32%)	3 (12%)	8 (32%)	27 (68%)	27 (68%)	26 (67%)	27 (68%)	9 (29%)	10 (30%)	6 (18%)	10 (30%)	18 (90%)	17 (85%)	14 (70%)	18 (90%)
Seg 2																
1	0	0	0	0	1 (2.5%)	1 (2.5%)	4 (10%)	1 (2.5%)	2 (6.5%)	1 (3.0%)	3 (9.1%)	1 (3.0%)	0 (0%)	0 (0%)	1 (5.0%)	0 (0%)
2	1 (4.0%)	1 (4.0%)	5 (20%)	1 (4.0%)	1 (2.5%)	0 (0%)	1 (2.5%)	0 (0%)	0 (0%)	1 (3.0%)	3 (9.1%)	0 (0%)	0	0	0	0
3	7 (28%)	7 (28%)	9 (36%)	7 (28%)	8 (20%)	8 (20%)	10 (25%)	9 (22%)	8 (26%)	11 (33%)	8 (24%)	11 (33%)	1 (5.0%)	1 (5.0%)	2 (10%)	1 (5.0%)
4	11 (44%)	11 (44%)	10 (40%)	11 (44%)	9 (22%)	9 (22%)	18 (45%)	9 (22%)	16 (52%)	15 (45%)	16 (48%)	16 (48%)	3 (15%)	3 (15%)	12 (60%)	2 (10%)
5	6 (24%)	6 (24%)	1 (4.0%)	6 (24%)	21 (52%)	22 (55%)	7 (18%)	21 (52%)	5 (16%)	5 (15%)	3 (9.1%)	5 (15%)	16 (80%)	16 (80%)	5 (25%)	17 (85%)
Seg 3																
1	0	0	0	0	2 (5.0%)	1 (2.5%)	2 (5.0%)	1 (2.5%)	2 (6.5%)	1 (3.0%)	3 (9.1%)	1 (3.0%)	0 (0%)	0 (0%)	2 (10%)	0 (0%)
2	1 (4.0%)	1 (4.0%)	8 (32%)	1 (4.0%)	1 (2.5%)	0 (0%)	3 (7.5%)	0 (0%)	0 (0%)	2 (6.1%)	3 (9.1%)	0 (0%)	0 (0%)	1 (5.0%)	0 (0%)	0 (0%)
3	7 (28%)	7 (28%)	6 (24%)	7 (28%)	8 (20%)	8 (20%)	8 (20%)	9 (22%)	8 (26%)	9 (27%)	10 (30%)	11 (33%)	0 (0%)	0 (0%)	1 (5.0%)	0 (0%)
4	11 (44%)	11 (44%)	10 (40%)	11 (44%)	8 (20%)	8 (20%)	15 (38%)	8 (20%)	16 (52%)	16 (48%)	14 (42%)	16 (48%)	4 (20%)	2 (10%)	10 (50%)	3 (15%)
5	6 (24%)	6 (24%)	1 (4.0%)	6 (24%)	21 (52%)	23 (57%)	12 (30%)	22 (55%)	5 (16%)	5 (15%)	3 (9.1%)	5 (15%)	16 (80%)	17 (85%)	7 (35%)	17 (85%)
Seg 4																
1	0	0	0	0	0 (0%)	0 (0%)	8 (20%)	0 (0%)	1 (3.2%)	1 (3.0%)	4 (12%)	1 (3.0%)	0 (0%)	0 (0%)	4 (20%)	0 (0%)
2	0 (0%)	0 (0%)	12 (48%)	0 (0%)	1 (2.5%)	1 (2.5%)	4 (10%)	1 (2.5%)	3 (9.7%)	2 (6.1%)	3 (9.1%)	1 (3.0%)	0 (0%)	0 (0%)	1 (5.0%)	0 (0%)
3	4 (16%)	5 (20%)	7 (28%)	4 (16%)	3 (7.5%)	2 (5.0%)	6 (15%)	2 (5.0%)	4 (13%)	6 (18%)	15 (45%)	7 (21%)	2 (10%)	0 (0%)	13 (65%)	0 (0%)
4	11 (44%)	10 (40%)	5 (20%)	11 (44%)	7 (18%)	7 (18%)	14 (35%)	8 (20%)	14 (45%)	14 (42%)	7 (21%)	14 (42%)	5 (25%)	6 (30%)	1 (5.0%)	6 (30%)
5	10 (40%)	10 (40%)	1 (4.0%)	10 (40%)	29 (72%)	30 (75%)	8 (20%)	29 (72%)	9 (29%)	10 (30%)	4 (12%)	10 (30%)	13 (65%)	14 (70%)	1 (5.0%)	14 (70%)
Seg 5–8																
1	0 (0%)	0 (0%)	1 (4.0%)	0 (0%)	1 (2.5%)	1 (2.5%)	2 (5.0%)	0 (0%)	1 (3.2%)	1 (3.0%)	4 (12%)	1 (3.0%)	0 (0%)	0 (0%)	4 (20%)	0 (0%)
2	0 (0%)	0 (0%)	11 (44%)	0 (0%)	3 (7.5%)	3 (7.5%)	5 (12%)	2 (5.0%)	3 (9.7%)	2 (6.1%)	3 (9.1%)	1 (3.0%)	5 (25%)	4 (20%)	7 (35%)	3 (15%)
3	4 (16%)	4 (16%)	7 (28%)	4 (16%)	5 (12%)	4 (10%)	8 (20%)	5 (12%)	4 (13%)	7 (21%)	14 (42%)	8 (24%)	11 (55%)	11 (55%)	8 (40%)	12 (60%)
4	11 (44%)	11 (44%)	5 (20%)	11 (44%)	7 (18%)	9 (22%)	16 (40%)	8 (20%)	14 (45%)	14 (42%)	8 (24%)	14 (42%)	1 (5.0%)	1 (5.0%)	1 (5.0%)	1 (5.0%)
5	10 (40%)	10 (40%)	1 (4.0%)	10 (40%)	24 (60%)	23 (57%)	9 (22%)	25 (62%)	9 (29%)	9 (27%)	4 (12%)	9 (27%)	3 (15%)	4 (20%)	0 (0%)	4 (20%)
Overall liver																
1	0	0	0	0	0 (0%)	0 (0%)	2 (5.0%)	0 (0%)	1 (3.2%)	1 (3.0%)	4 (12%)	1 (3.0%)	0 (0%)	0 (0%)	1 (5.0%)	0 (0%)
2	0 (0%)	0 (0%)	9 (36%)	0 (0%)	0 (0%)	0 (0%)	3 (7.5%)	0 (0%)	2 (6.5%)	1 (3.0%)	3 (9.1%)	0 (0%)	0 (0%)	0 (0%)	4 (20%)	0 (0%)
3	5 (20%)	6 (24%)	11 (44%)	5 (20%)	5 (12%)	5 (12%)	11 (28%)	5 (12%)	5 (16%)	8 (24%)	11 (33%)	9 (27%)	4 (20%)	3 (15%)	7 (35%)	1 (5.0%)
4	12 (48%)	11 (44%)	5 (20%)	12 (48%)	15 (38%)	14 (35%)	21 (52%)	15 (38%)	14 (45%)	14 (42%)	10 (30%)	14 (42%)	9 (45%)	10 (50%)	8 (40%)	11 (55%)
5	8 (32%)	8 (32%)	0 (0%)	8 (32%)	20 (50%)	21 (52%)	3 (7.5%)	20 (50%)	9 (29%)	9 (27%)	5 (15%)	9 (27%)	7 (35%)	7 (35%)	0 (0%)	8 (40%)
Spleen																
4	0	0	0	0	0	0	0	0	0	0	0	1 (3.0%)	0	0	0	0
5	0	0	0	25 (100%)	0	0	0	40 (100%)	0	0	0	32 (97%)	0	0	0	20 (100%)

In ${{\text{M}}}_{{\text{seg}}+{\text{spleen}}}$ vs.${{\text{M}}}_{{\text{nnU}}-{\text{Net}}}$, ${{\text{M}}}_{{\text{seg}}+{\text{spleen}}}$ showed DSC values of 0.89 (mean) and 0.91 (average of median) which are similar to mean DSC of 0.89 and average of median DSC of 0.92 of ${{\text{M}}}_{{\text{nnU}}-{\text{Net}}}$. Individual DSC difference between (${{\text{M}}}_{{\text{seg}}+{\text{spleen}}}- {{\text{M}}}_{{\text{nnU}}-{\text{Net}}}$) were 0 for all segments except the median of segment 4 where ${{\text{M}}}_{{\text{nnU}}-{\text{Net}}}$>${{\text{M}}}_{{\text{seg}}+{\text{spleen}}}$ by 0.01. ${{\text{f}}}_{{{\text{M}}}_{{{\text{seg+spleen}}}}:{{\text{M}}}_{{\text{nnU-Net}}}}$ was negligible or 1:1) (see Table S4 for details). The difference in the mean and median ${{\text{HD}}}_{{\text{95}}}$ of the two models were negligible (range =  − 0.62 to 0.01 mm). Lastly, the difference in the mean and median PDV of the two models ranged from -1.7% to 0.6%. Qualitatively, the difference between percent of cases receiving score $\ge$ 3 across two models were within 1% except segment 5–8 where ${{\text{M}}}_{{\text{seg}}+{\text{spleen}}}$ led by 5%. Overall, the results from two models were equivalent.

Lastly, the Wilcoxon signed-rank test showed that ${{\text{M}}}_{{\text{seg}}+{\text{spleen}}}$ and ${{\text{M}}}_{{\text{nnU}}-{\text{Net}}}$ were not significantly different in their metrics (p > 0.05). Comparison of ${{\text{M}}}_{{\text{seg}}+{\text{spleen}}}$ with ${{\text{M}}}_{{\text{vess}}}$ showed no significance in most cases with few exceptions (see footer of Table 7). Furthermore, ${{\text{M}}}_{{\text{seg}}+{\text{spleen}}}$ showed better agreement than ${{\text{M}}}_{{\text{vess}}}$ in terms of ${{\text{HD}}}_{{\text{95}}}$. Therefore, in overall comparison, we establish that ${{\text{M}}}_{{\text{seg}}+{\text{spleen}}}\sim {{\text{M}}}_{{\text{nnU}}-{\text{Net}}}$ and ${{\text{M}}}_{{\text{nnU}}-{\text{Net}}} >{{\text{M}}}_{{\text{vess}}}$. We selected ${{\text{M}}}_{{\text{seg}}+{\text{spleen}}}$ as our best model due to its wider application as the mean/median DSC of spleen is 0.99.

#### Assessment of the models on the liver surgery patients

Table 7 shows the results from quantitative assessment of our models on the pre-surgery CTs. The mean and average of median DSC values of ${{\text{M}}}_{{\text{seg}}+{\text{spleen}}}$ across all segments were 0.91 and 0.92, respectively, and those for spleen were 0.91 and 0.96. Individually, the mean and median DSCs of all segments from ${{\text{M}}}_{{\text{seg}}+{\text{spleen}}}$ were $\ge 0.90$ except segment 2 where median and mean DSC were 0.86 and 0.85, respectively. With regards to distance metrics, segment 2 from ${{\text{M}}}_{{\text{seg}}+{\text{spleen}}}$ showed a mean and median ${{\text{HD}}}_{95}$ values of 8.5 mm and 9.4 mm which was the highest among all other segments. The best ${{\text{HD}}}_{95}$ were obtained in case of segment 1 with mean and median values of 2.8 mm and 3.2 mm. Additionally, spleen showed mean ${{\text{HD}}}_{95}$ of 2.2 mm. With regard to volumetric comparison, ${{\text{M}}}_{{\text{seg}}+{\text{spleen}}}$ vs radiologist ground-truth contours, the overall mean and average median values across all segments were 8.2% and 5.6%. Likewise, mean and median PDV for spleen were within 2%. Lastly, stratification of DSC based on the cancer type showed no performance change in segments ($\pm$ 1%) but spleen of CC (N = 5) showed 2% lesser DSC than CRM (N = 22) cases.

**Table 7:** Comparison of descriptive statistics from models trained with segments, segments with vessel (color channel) and segments with spleen on liver surgery cohort C_LS_.

	Seg 1,				Seg 2,				Seg 3,				Seg 4,				Seg 5–8,				Spleen,
	N = 33^1^				N = 33^1^				N = 33^1^				N = 33^1^				N = 33^1^				N=33^1^
	(IntraMD^2a^ = 0.88)				(IntraMD^2a^ = 0.88)				(IntraMD^2a^ = 0.94)				(IntraMD^2a^ = 0.92)				(IntraMD^2a^ = 0.99)﻿				
	(InterMD^2b^ = 0.82)				(InterMD^2b^ = 0.85)				(InterMD^2b^ = 0.91)				(InterMD^2b^ = 0.88)				(InterMD^2b^ = 0.96)﻿				
	M_paU-Net_^1^	M_nnUnet_^1^	M_seg+spleen_^1^	M_vess_^1^	M_paU-Net_^1^	M_nnUnet_^1^	M_seg+spleen_^1^	M_vess_^1^	M_paU-Net_^1^	M_nnUnet_^1^	M_seg+ spleen_^1^	M_vess_^1^	M_paU-Net_^1^	M_nnUnet_^1^	M_seg+_ _spleen_^1^	M_vess_^1^	M_paU-Net_^1^	M_nnUnet_^1^	M_seg+spleen_^1^	M_vess_^1^	M_seg+spleen_^1^
Dice	0.91	0.94	0.94	0.94	0.87	0.88	0.86	0.86	0.90	0.93	0.91	0.91	0.89	0.93	0.92	0.93	0.97	0.98	0.98	0.98	0.96
	0.85,0.17	0.92,0.06	0.92,0.07	0.86, 0.23	0.85, 0.06	0.86, 0.06	0.85, 0.07	0.80, 0.22	0.89, 0.05	0.91, 0.04	0.91, 0.04	0.85, 0.22	0.86, 0.09	0.91, 0.06	0.90, 0.07	0.86, 0.23	0.96, 0.03	0.98, 0.02	0.98, 0.02	0.91, 0.24	0.91, 0.23
	0.96,0.00	0.98,0.75	0.98,0.69	0.98, 0.00	0.93, 0.62	0.94, 0.63	0.94, 0.62	0.93, 0.00	0.95, 0.69	0.96, 0.75	0.96, 0.76	0.96, 0.00	0.96, 0.58	0.97, 0.72	0.96, 0.63	0.97, 0.00	0.99, 0.88	0.99, 0.92	0.99, 0.91	0.99, 0.00	0.98, 0.00
${{\text{HD}}}_{95}^{3}$	4	2.5	2.8	2.5	8	7.8	8.5	6.8	7	5.4	5.8	5.8	10	5.0	5.8	5.0	5	2.5	3.0	2.2	2.1
	14, 2	3.0, 1.6	3.2, 2.0	3.0, 1.9	9, 5	8.1, 3.2	9.4, 4.1	7.9, 4.8	10, 10	8.7, 14.8	6.6, 3.0	6.4, 4.1	12, 8	6.8, 5.2	7.4, 5.0	6.2, 4.6	9, 12	4.1, 3.8	4.6, 4.3	5.5, 9.9	2.2, 0.8
	229, 2	6.4, 0.9	8.3, 0.8	8.0, 0.0	23, 3	15.7, 2.8	22.3, 3.0	23.4, 0.0	55, 3	89.8, 2.5	15.4, 2.7	22.2, 0.0	36, 3	23.2, 1.7	18.9, 2.5	16.1, 0.0	65, 1	16.2, 0.8	19.8, 0.8	51.6, 0.0	5.2, 1.3
Avg.${{\text{HD}}}_{{\text{A}}}^{3}$	0.20	0.11	0.10	0.11	0.38	0.39	0.50	0.40	0.31	0.22	0.26	0.23	0.42	0.16	0.21	0.17	0.09	0.03	0.04	0.03	0.05
	6.70,35.86	0.17,0.16	0.19,0.21	0.18, 0.19	0.59, 0.40	0.51, 0.37	0.62, 0.45	0.57, 0.63	0.51, 0.63	0.52, 1.47	0.32, 0.21	0.32, 0.34	0.79, 0.95	0.31, 0.37	0.37, 0.46	0.29, 0.32	0.21, 0.37	0.06, 0.08	0.07, 0.07	0.17, 0.52	0.05, 0.03
	206.36,0.06	0.74,0.02	0.98,0.02	0.74, 0.00	1.77, 0.16	1.90,0.12	2.15, 0.15	2.96, 0.00	3.52, 0.10	8.63, 0.06	0.93, 0.10	1.86, 0.00	3.54, 0.08	1.25, 0.04	1.97, 0.05	1.24, 0.00	2.08, 0.01	0.37, 0.01	0.28, 0.01	2.83, 0.00	0.17, 0.02
PDV	12	4	3.8	3	12	11	12.0	9	5	4	5.8	4	6	5	4.6	4	3	2	1.9	1	1.3
	17, 23	6, 5	6.2, 5.7	6, 6	15, 15	14, 16	15.3, 18.8	14, 20	7, 8	7, 7	6.8, 4.2	7, 8	14, 21	9, 13	10.6, 15.2	7, 11	4, 3	2, 2	2.3, 2.0	3, 5	1.9, 1.8
	133, 0	24, 0	26.7, 0.0	26, 0	89, 1	95, 0	110.3, 0.9	118, 0	36, 0	35, 1	18.0, 0.5	37, 0	92, 0	54, 0	64.9, 0.7	47, 0	15, 0	9, 0	9.2, 0.1	25, 0	7.6, 0.0

^1^Data in each cell is organized as row 1 = Median, row 2 = Mean, Standard deviation, row 3 = Max, Min; ^1^M_nnUnet_ = model trained with segments only, M_seg+spleen_ = Model trained with Segments and Spleen as labels, M_vess_ = Model trained with segments as label and vessel as color channel; ^2a^IntraMD = Intra-observer mean dice,^2b^IntrerMD = Inter-observer mean dice, ^3^HD = Hausdorff distance (95 = 95th percentile and A = Average) in mm; PDV = percent difference in volume.

In comparison to ${{\text{M}}}_{{\text{seg}}+{\text{spleen}}}$, ${{\text{M}}}_{{\text{paU}}-{\text{Net}}}$ and ${{\text{M}}}_{{\text{vess}}}$ showed poor performance in case of segment 1 as mean DSC of ${{\text{M}}}_{{\text{seg}}+{\text{spleen}}}$ were greater than other two models by 7% and 6%, respectively. On segment 3 and 4, ${{\text{M}}}_{{\text{seg}}+{\text{spleen}}}$ outperformed ${{\text{M}}}_{{\text{paU}}-{\text{Net}}}$ by 2% and 5%, respectively. Moreover, the mean DSC value of ${{\text{M}}}_{{\text{Vess}}}$ was around 5% less that other models on segments 2, 3, and 4 which supports that vessels architectures and segments 2 and 3 boundary are sensitive to each other. The mean DSC of other three models were within 2% of one another. With regards to HD, ${{\text{M}}}_{{\text{paU}}-{\text{Net}}}$ showed the largest HD but all other models showed similar performance.

Qualitatively, regarding ${{\text{M}}}_{{\text{seg}}+{\text{spleen}}}$, 97% of segments showed a score $\ge 3$ with 69% showing a score of $\ge 4$ and 27% showing a score of 5. Individually, at least 64% of each segment showed a score of 4 or more. Segments 1, 4, and 5–8 received higher scores than segments 2 which is highlighted by the lower value of 15% (score of 5) in Table 5. Compared with other models, contours from ${{\text{M}}}_{{\text{seg}}+{\text{spleen}}}$ included 14% more cases of Likert score $\ge 3$ than ${{\text{M}}}_{{\text{paU}}-{\text{Net}}}$. However, the other two models received similar scores as the ${{\text{M}}}_{{\text{seg}}+{\text{spleen}}}$.

#### Assessment of the models on challenge datasets

Tables 6 and 8 shows the results from quantitative and qualitative assessment of all models on the challenge dataset (C_CH_). With regards to the best model (${{\text{M}}}_{{\text{Seg}}+{\text{Spleen}}}$), both overall mean and median DSC values of segments were 0.87. The individual mean and median DSC values were $\ge$ 0.96 for segment 5–8 and spleen whereas the mean/median DSC for segments 1, 2, 3, 4 ranged 0.80 to 0.88. Segment 2 had the lowest mean and median DSCs of 0.80. For distance metrics, segment 1 and spleen had a mean and median ${{\text{HD}}}_{95}$ within 5 mm which was better than all other segments. The largest mean and median ${{\text{HD}}}_{95}$ values were $\ge 10$ mm which was observed in the segment 2. Lastly, the overall mean and average median PDV were 11% and 9.5% for segment and 2% for spleen. Largest PDVs were observed in segment 2 with mean/median of 19%. Lastly, since the cancer types of challenge datasets are not available, we could not perform stratified DSC analysis. Comparatively, both ${{\text{M}}}_{{\text{nnU}}-{\text{Net}}}$ and ${{\text{M}}}_{{\text{Vess}}}$ showed mean and median DSC/HD_95_/PDV within 1%/1 mm/2% of our best model. On the other hand, ${{\text{M}}}_{{\text{paU}}-{\text{Net}}}$ showed mean and median HD_95_/PDV of 5 mm/6% higher than the that of the best model.

**Table 8:** Comparison of descriptive statistics from models trained with segments, segments with vessel (color channel) and segments with spleen on challenge cohort C_CH_.

	Seg 1, N = 25^1^				Seg 2, N = 25^1^				Seg 3, N = 25^1^				Seg 4, N = 25^1^				Seg 5–8, N = 25^1^				Spleen, N = 25^1^
	(IntraMD^2a^ = 0.88)				N = 33^1^				(IntraMD^2a^ = 0.94)				(IntraMD^2a^ = 0.92)				(IntraMD^2a^ = 0.99)﻿				
	(InterMD^2b^ = 0.82)				(InterMD^2b^ = 0.85)				(InterMD^2b^ = 0.91)				(InterMD^2b^ = 0.88)				(InterMD^2b^ = 0.96)﻿				
	M_paU-Net_^1^	M_nnUnet_^1^	M_seg+spleen_^1^	M_vess_^1^	M_paU-Net_^1^	M_nnUnet_^1^	M_seg+spleen_^1^	M_vess_^1^	M_paU-Net_^1^	M_nnUnet_^1^	M_seg+spleen_^1^	M_vess_^1^	M_paU-Net_^1^	M_nnUnet_^1^	M_seg+spleen_^1^	M_vess_^1^	M_paU-Net_^1^	M_nnUnet_^1^	M_seg+spleen_^1^	M_vess_^1^	M_seg+spleen_^1^
Dice	0.87	0.88	0.88	0.88	0.80	0.80	0.80	0.81	0.83	0.85	0.85	0.86	0.80	0.85	0.85	0.84	0.96	0.97	0.97	0.96	0.95
	0.85,0.05	0.88,0.04	0.88,0.04	0.87,0.05	0.79, 0.05	0.80, 0.05	0.80, 0.05	0.81, 0.04	0.83, 0.06	0.85, 0.05	0.85, 0.05	0.85, 0.06	0.80, 0.07	0.85, 0.06	0.84, 0.06	0.84, 0.06	0.96, 0.01	0.97, 0.01	0.97, 0.01	0.96, 0.01	0.96, 0.01
	0.94,0.72	0.97,0.77	0.96,0.76	0.97,0.77	0.88, 0.67	0.88, 0.65	0.89, 0.65	0.87, 0.68	0.92, 0.67	0.94, 0.67	0.95, 0.70	0.94, 0.65	0.93, 0.66	0.95, 0.74	0.95, 0.73	0.94, 0.72	0.99, 0.92	0.99, 0.95	0.99, 0.95	0.99, 0.94	0.99, 0.93
${{\text{HD}}}_{95}^{3}$	5	3.8	3.8	4.3	10	9.8	10.0	10.7	10	9.1	8.8	9.0	14	8.6	8.4	8.9	8	6.1	6.1	6.0	2.0
	6, 3	4.1, 2.3	4.2, 2.6	4.4, 2.3	12, 5	11.5, 5.0	11.4, 5.1	12.0, 5.9	11, 4	9.3, 3.2	9.5, 3.2	9.5, 3.0	16, 9	10.5, 4.9	10.8, 5.2	10.5, 5.0	9, 6	7.3, 5.2	7.6, 5.7	7.3, 4.8	1.9, 0.5
	15, 2	12.8, 0.7	14.1, 0.7	13.0, 0.7	25, 5	25.9, 5.6	25.2, 5.5	29.3, 5.6	24, 6	15.9, 4.9	16.0, 5.0	16.6, 4.6	43, 4	23.4, 3.1	24.0, 3.2	21.9, 3.4	26, 2	22.8, 1.4	23.2, 1.4	21.9, 1.0	2.8, 0.7
Avg.${{\text{HD}}}_{{\text{A}}}^{3}$	0.26	0.21	0.21	0.22	0.76	0.71	0.69	0.74	0.58	0.45	0.49	0.47	1.17	0.52	0.54	0.45	0.15	0.10	0.09	0.09	0.06
	0.35, 0.30	0.25, 0.20	0.25,0.22	0.27,0.20	0.88, 0.53	0.81, 0.46	0.81, 0.47	0.83, 0.45	0.73, 0.52	0.56, 0.33	0.55, 0.27	0.58, 0.37	1.06, 0.64	0.63, 0.48	0.66, 0.51	0.64, 0.47	0.19, 0.14	0.11, 0.09	0.12, 0.11	0.11, 0.09	0.06, 0.02
	1.42, 0.06	1.10, 0.02	1.21,0.03	1.08,0.02	2.19, 0.24	2.14, 0.30	2.28, 0.41	1.91, 0.36	1.99, 0.23	1.61, 0.13	1.30, 0.12	1.95,0.18	2.49, 0.12	2.07, 0.07	2.12, 0.07	1.86, 0.08	0.51, 0.01	0.43, 0.01	0.44, 0.01	0.40, 0.01	0.10, 0.00
PDV	14	8	8	11	18	16	18	18	13	9	8	8	9	7	8	6	2	2	3	3	4
	17, 12	11, 8	11, 10	12, 8	19, 12	19, 13	19, 11	19, 11	14, 13	12, 9	12, 10	11, 10	11, 8	10, 7	10, 8	9, 8	2, 2	3, 2	3, 2	3, 2	4, 2
	52, 1	31, 0	34, 0	31, 1	44, 1	49, 0	50, 2	47, 2	46, 0	37, 1	40, 0	37, 0	26, 0	30, 0	33, 0	30, 0	6, 0	7, 0	7, 0	8, 0	8, 0

Qualitatively, 100% of cases in ${{\text{M}}}_{{\text{seg}}+{\text{spleen}}}$ received an overall Likert score $\ge$ 3 with more than 80% received score $\ge$ 4. Lower Likert scores were localized to segments 2 and 3 contours. Regarding other models, Likert scores of ${{\text{M}}}_{{\text{Vess}}}$ and ${{\text{M}}}_{{\text{Seg}}}$ showed similar trend as the ${{\text{M}}}_{{\text{Seg}}+{\text{Spleen}}}$. In contrast, the percentage of cases of ${{\text{M}}}_{{\text{paU}}-{\text{Net}}}$ receiving score $\ge$ 3 was 64% with only 20% showing overall score $\ge$ 4. Lastly, more than 97% of spleen from ${{\text{M}}}_{{\text{seg}}+{\text{spleen}}}$ received score $\ge$ 4.

#### Assessment of the models on post-portal vein embolization images

As per Table 9, ${{\text{M}}}_{{\text{Seg}}+{\text{Spleen}}}$ showed mean and median DSCs ≥ 0.87 for all segments and spleen except segment 2 where the mean and median DSCs were 0.82 and 0.80 in the case of segment 2. Furthermore, segments 2, 3, 4, 5–8, showed mean/median DSCs ${{\text{HD}}}_{95}$≥7 mm. Segment 1 and Spleen showed mean/median ${{\text{HD}}}_{95}$ within 5 mm. Mean and median PDVs of segments 1, 3, 4, 5–8 were within 10% but that of segment 2 was ≥ 15%. The stratified DSC analysis using cancer types showed CC (N = 3) larger DSC CRM (N = 17) by 2 to 4%. With regards to other models, all of the models showed mean and median DSCs within 1% of ${{\text{M}}}_{{\text{Seg}}+{\text{Spleen}}}$ with the exception of ${{\text{M}}}_{{\text{paU}}-{\text{Net}}}$ in case of segment 4 where DSCs were less than that of ${{\text{M}}}_{{\text{Seg}}+{\text{Spleen}}}$ by 7%. Similar trends were observed in ${{\text{HD}}}_{95}$ with the exception of ${{\text{M}}}_{{\text{paU}}-{\text{Net}}}$ showing mean ${{\text{HD}}}_{95}$ up to 18% in the case of segment 5–8. Except ${{\text{M}}}_{{\text{paU}}-{\text{Net}}}$, PDVs of all models were within 4% of one another. Mean PDVs of ${{\text{M}}}_{{\text{paU}}-{\text{Net}}}$ were greater than that of ${{\text{M}}}_{{\text{Seg}}+{\text{Spleen}}}$ by 16%.

**Table 9:** Comparison of descriptive statistics from models trained with segments, segments with vessel (color channel) and segments with spleen on portal vein embolization cohort C_PVE_.

	Seg 1, N = 20				Seg 2, N = 20^1^				Seg 3, N = 20^1^				Seg 4, N = 20^1^				Seg 5–8, N = 20^1^				Spleen, N = 20^1^
	(IntraMD^2a^ = 0.88)				(IntraMD^2a^ = 0.88)				(IntraMD^2a^ = 0.94)				(IntraMD^2a^ = 0.92)				(IntraMD^2a^ = 0.99)				
	(InterMD^2b^ = 0.82)				(InterMD^2b^ = 0.85)				(InterMD^2b^ = 0.91)				(InterMD^2b^ = 0.88)				(InterMD^2b^ = 0.96)				
	M_paU-Net_^1^	M_nnUnet_^1^	M_seg+spleen_^1^	M_vess_^1^	M_paU-Net_^1^	M_nnUnet_^1^	M_seg+spleen_^1^	M_vess_^1^	M_paU-Net_^1^	M_nnUnet_^1^	M_seg+spleen_^1^	M_vess_^1^	M_paU-Net_^1^	M_nnUnet_^1^	M_seg+spleen_^1^	M_vess_^1^	M_paU-Net_^1^	M_nnUnet_^1^	M_seg+spleen_^1^	M_vess_^1^	M_seg+spleen_^1^
Dice	0.90	0.90	0.91	0.90	0.81	0.82	0.82	0.82	0.87	0.88	0.88	0.88	0.85	0.89	0.89	0.89	0.92	0.93	0.93	0.94	0.97
	0.88, 0.05	0.88, 0.06	0.88, 0.06	0.89,0.05	0.77, 0.19	0.80, 0.10	0.80, 0.10	0.80, 0.11	0.86, 0.05	0.87, 0.05	0.87, 0.05	0.87, 0.05	0.81, 0.10	0.88, 0.05	0.88, 0.05	0.87, 0.07	0.91, 0.06	0.92, 0.04	0.93, 0.04	0.92, 0.05	0.97, 0.01
	0.95, 0.75	0.94, 0.69	0.94, 0.67	0.94, 0.75	0.89, 0.00	0.89, 0.42	0.89, 0.43	0.89, 0.41	0.92, 0.69	0.93, 0.68	0.92, 0.71	0.93, 0.70	0.91, 0.46	0.94, 0.77	0.95, 0.75	0.94, 0.64	0.96, 0.72	0.98, 0.82	0.98, 0.84	0.98, 0.80	0.98, 0.96
${{\text{HD}}}_{95}^{3}$	4	4.3	4	4	13	10.9	11	11	12	9.3	10	9	15	7.8	8	9	15	14.9	11	10	2
	5, 4	5.5, 4.3	5, 4	5, 3	16, 13	13.5, 10.3	12, 6	14, 11	17, 14	10.2, 4.3	11, 8	12, 8	18, 8	9.5, 5.1	9, 6	11, 7	18, 10	16.1, 15.9	12, 7	12, 9	2, 0
	16, 2	21.7, 2.5	21, 2	17, 2	66, 5	51.7, 5.8	27, 6	55, 5	56, 5	22.0, 5.8	40, 5	42, 5	36, 6	21.9, 3.4	22, 3	27, 3	47, 5	77.2, 2.5	27, 2	41, 3	3, 1
Avg.${{\text{HD}}}_{{\text{A}}}^{3}$	0.21	0.19	0.19	0.21	0.80	0.72	0.70	0.71	0.53	0.43	0.46	0.45	0.89	0.36	0.37	0.43	0.28	0.30	0.26	0.23	0.03
	0.33,0.32	0.35,0.47	0.35,0.49	0.31, 0.31	2.35,6.49	0.96, 0.84	0.96, 0.78	1.14, 1.43	0.91, 0.87	0.60, 0.60	0.65, 0.77	0.68, 0.84	1.15,0.92	0.49, 0.38	0.50, 0.45	0.62, 0.65	0.61, 0.94	0.42, 0.36	0.33, 0.30	0.41, 0.51	0.03, 0.01
	1.26,0.06	2.15, 0.09	2.26, 0.08	1.43, 0.08	29.81,0.23	3.71, 0.27	3.15, 0.25	6.47, 0.26	3.56,0.20	2.92, 0.21	3.73, 0.20	4.11, 0.20	3.41, 0.24	1.49, 0.10	1.74, 0.07	2.83, 0.09	4.19, 0.09	1.25, 0.03	1.16, 0.03	2.26, 0.04	0.08, 0.02
PDV	7	6	6	5	19	15	15	20	7	5	6	6	13	6	7	6	3	4	4	4	1
	7, 5	10, 18	10, 20	8, 13	34, 68	18, 13	18, 13	22, 16	9, 6	7, 6	7, 7	8, 6	14, 14	8, 7	9, 8	10, 12	6, 5	6, 6	6, 5	6, 6	1, 1
	19, 0	85, 0	92, 0	61, 1	320, 3	53, 1	53, 5	56, 0	23, 2	22, 0	25, 1	26, 1	62, 1	26, 0	32, 1	48, 0	16, 0	24, 1	22, 0	22, 0	3, 0

^1^Data in each cell is organized as row 1 = Median, row 2 = Mean, Standard deviation, row 3 = Max, Min; ^1^M_nnUnet_ = model trained with segments only, M_seg+spleen_ = Model trained with Segments and Spleen as labels, M_vess_ = Model trained with segments as label and vessel as color channel; ^2a^IntraMD = Intra-observer mean dice,^2b^IntrerMD = Inter-observer mean dice, ^3^HD = Hausdorff distance (95 = 95th percentile and A = Average) in mm; PDV = percent difference in volume.

**Figure 2:**
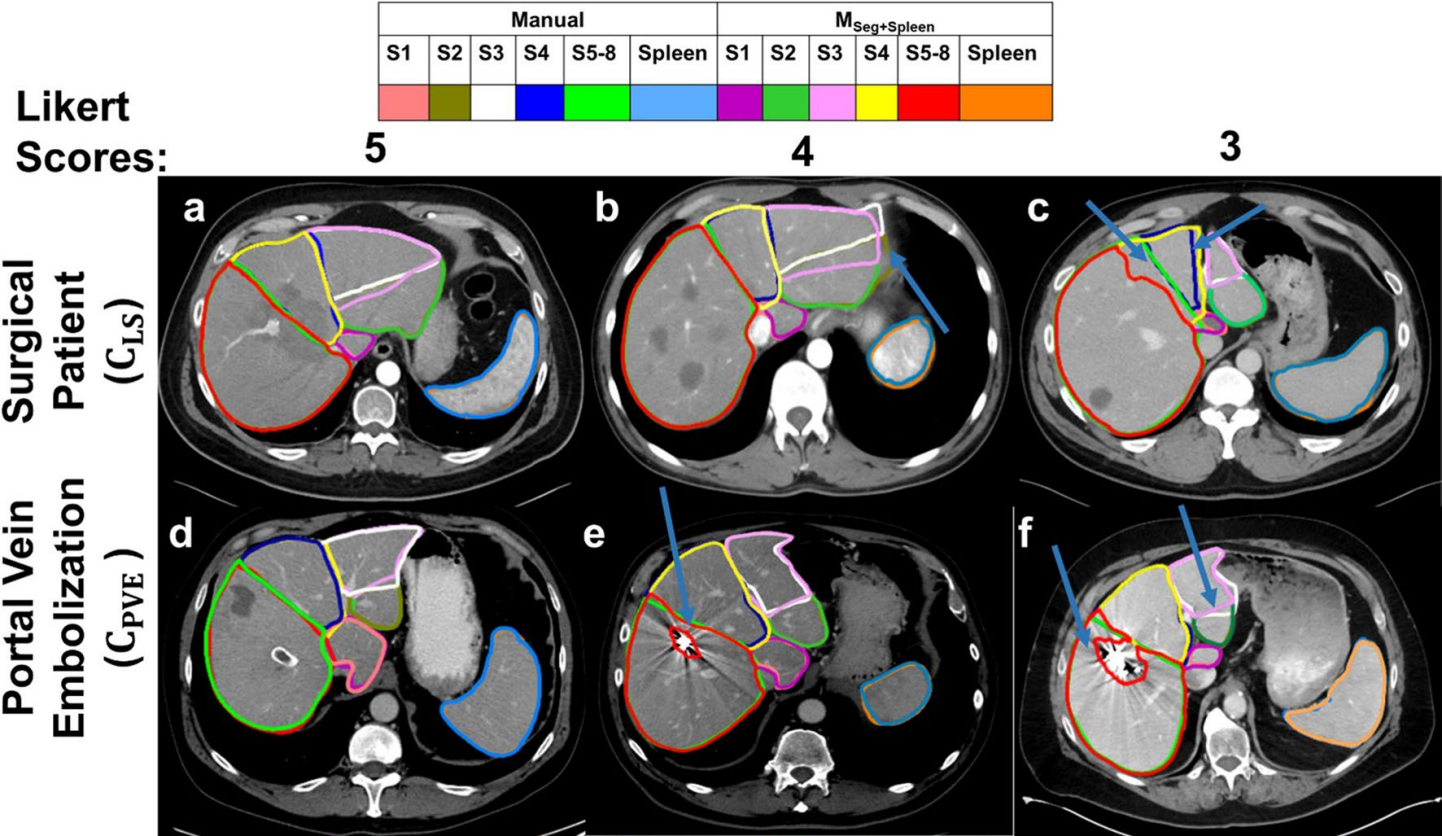
Example cases of three different Likert score (5, 4, and 3) is shown for two different cohorts. Blue arrow highlights the uncertainties in boundaries between the manual and model predicted contours. For score 4 and 3 in the images of ${{\text{C}}}_{{\text{PVE}}}$, the arrow highlights the hole in segment 5–8 due to metal artifacts. In ${{\text{C}}}_{{\text{PVE}}}$, a score of 4 is given when image has a hole, but segments boundaries follow the vessels.

Qualitatively, at least 90% of cases received a score $\ge 3$ and at least 85% received a score of $\ge 4$ across all models in each segment with the exception of segment 4 and 5–8. For segments 4 and 5–8, only 5% and 10% cases of ${{\text{M}}}_{{\text{paU}}-{\text{Net}}}$ received score $\ge$ 4 whereas at least 25% cases of ${{\text{M}}}_{{\text{Seg}}+{\text{Spleen}}}$ received score $\ge$ 4. Additionally, a score ≥ 3 was received by more than 45% of cases of segment 5–8 across all models. Lastly, all cases of spleen received a score of 5. Examples of Likert scores with the specific images are shown in Fig. 2.

#### Assessment of the model on non-contrast images

As per Table 10, ${{\text{M}}}_{{\text{Seg}}+{\text{Spleen}}}$ showed mean and median DSCs $\ge$ 0.83 across all segments and spleen with the exception of segment 1 and 2 where the mean DSCs were 0.70 and 0.78 respectively. Further, mean, and median ${{\text{HD}}}_{95}$ were $\ge 5{\text{mm}}$ across all segments but spleen showed ${{\text{HD}}}_{95}$ <5 mm. Segment 1 showed a mean and median PDVs of 18% and 30% which was the largest PDV compared to other segments. Next, The mean MDA ranged from 1.6–3.6 mm for segments and was 1.3 mm for spleen.

**Table 10:** Comparison of descriptive statistics from models trained with segments, segments with vessel (color channel) and segments with spleen on non-contrast image cohort C_NC_.

	Seg 1, N = 20 (IntraMD^2a^ = 0.88) (InterMD^2b^ = 0.82)			Seg 2, N = 20^1^ (IntraMD^2a^ = 0.88) (InterMD^2b^ = 0.85)			Seg 3, N = 20^1^ (IntraMD^2a^ = 0.94) (InterMD^2b^ = 0.91)			Seg 4, N = 20^1^ (IntraMD^2a^ = 0.92) (InterMD^2b^ = 0.88)			Seg 5–8, N = 20^1^ (IntraMD^2a^ = 0.99) (InterMD^2b^ = 0.96)			Spleen, N = 20^1^
	M_paU-Net_^1^	M_nnUnet_^1^	M_seg+spleen_^1^	M_paU-Net_^1^	M_nnUnet_^1^	M_seg+spleen_^1^	M_paU-Net_^1^	M_nnUnet_^1^	M_seg+spleen_^1^	M_paU-Net_^1^	M_nnUnet_^1^	M_seg+spleen_^1^	M_paU-Net_^1^	M_nnUnet_^1^	M_seg+spleen_^1^	M_seg+spleen_^1^
Dice	0.83	0.81	0.81	0.82	0.79	0.77	0.88	0.89	0.87	0.83	0.85	0.84	0.95	0.96	0.95	0.96
	0.78, 0.15	0.68, 0.26	0.70, 0.24	0.78, 0.11	0.78, 0.09	0.78, 0.09	0.87, 0.06	0.87, 0.06	0.87, 0.05	0.81, 0.10	0.83, 0.08	0.83, 0.08	0.94, 0.05	0.94, 0.03	0.94, 0.03	0.94, 0.06
	0.94, 0.35	0.89, 0.00	0.89, 0.06	0.92, 0.49	0.89, 0.59	0.90, 0.62	0.94, 0.75	0.95, 0.76	0.94, 0.76	0.96, 0.57	0.94, 0.60	0.94, 0.59	0.99, 0.76	0.97, 0.86	0.97, 0.86	0.97, 0.72
${{\text{HD}}}_{95}^{3}$	7	9	9.0	7	8	9.5	6	7	7.2	9	9	8.0	5	6	5.9	2.5
	21, 32	11, 8	10.6, 5.2	11, 8	12, 10	12.8, 9.8	7, 6	8, 3	7.5, 2.9	16, 22	10, 4	9.9, 4.3	7, 5	8, 6	6.6, 3.1	5.0, 4.9
	96, 4	32, 4	22.8, 4.8	39, 4	44, 6	44.1, 4.9	29, 3	18, 5	15.1, 4.0	107, 4	17, 3	18.1, 2.9	23, 1	29, 2	13.5, 2.8	15.9, 2.0
Avg.${{\text{HD}}}_{{\text{A}}}^{3}$	0.53	0.57	0.58	0.56	0.63	0.81	0.28	0.33	0.36	0.62	0.55	0.56	0.12	0.14	0.13	0.07
	1.53, 2.85	1.35, 1.53	1.31, 1.39	0.88, 0.75	0.97, 0.86	1.02, 0.86	0.38, 0.29	0.42, 0.27	0.41, 0.26	0.94, 0.97	0.66, 0.51	0.65, 0.49	0.22, 0.34	0.24, 0.24	0.18, 0.13	0.23, 0.39
	12.54, 0.16	5.75, 0.26	5.07, 0.27	2.89, 0.19	3.91, 0.32	3.74, 0.28	1.23, 0.12	1.13, 0.12	1.13, 0.13	3.37, 0.11	2.40, 0.10	2.25, 0.10	1.60, 0.03	1.10, 0.04	0.43, 0.04	1.57, 0.03
MDA	2.13	2.48	2.71	2.06	2.47	2.66	1.67	1.80	1.79	2.52	2.71	2.45	1.47	1.50	1.53	0.96
	3.66, 4.79	3.48, 2.60	3.59, 2.56	2.50, 1.11	3.00, 1.70	3.19, 1.83	1.95, 0.90	2.07, 0.67	2.08, 0.69	3.24, 2.15	2.75, 1.07	2.75, 1.08	1.69, 1.15	1.84, 0.81	1.64, 0.62	1.34, 0.95
	22.68, 0.89	10.46, 0.97	12.62, 1.33	5.26, 1.22	8.56, 1.62	8.29, 1.55	5.11, 1.14	3.46, 1.27	3.56, 1.11	9.46, 0.96	5.79, 1.04	5.68, 1.25	6.00, 0.48	3.55, 0.89	3.15, 0.82	4.40, 0.59
PDV	9	18	18	15	13	12	6	5	5	9	7	10	3	4	4	5
	23, 31	27, 25	30, 29	21, 23	19, 16	19, 18	8, 8	8, 8	7, 6	19, 19	8, 6	10, 6	6, 11	5, 5	5, 5	7, 6
	124, 0	81, 1	97, 1	103, 1	51, 0	55, 0	22, 1	30, 1	17, 1	61, 1	20, 1	23, 0	51, 0	19, 0	17, 0	22, 1

^1^Data in each cell is organized as row 1 = Median, row 2 = Mean, Standard deviation, row 3 = Max, Min; ^1^M_nnUnet_ = model trained with segments only, M_seg+spleen_ = Model trained with Segments and Spleen as labels, M_vess_ = Model trained with segments as label and vessel as color channel.

^2a^IntraMD = Intra-observer mean dice.

^2b^InterMD = Inter-observer mean dice.

^3^HD = Hausdorff distance (95 = 95th percentile and A = Average) in mm, MDA = Mean Distance to Agreement in mm, PDV = percent difference in volume.

${{\text{M}}}_{{\text{nnU}}-{\text{Net}}}$, showed similar performance as ${{\text{M}}}_{{\text{Seg}}+{\text{Spleen}}}$, across all metrics in all segments. Specifically, the agreement between the models were within 2%, 1.5 mm, and 3% and 0.2 mm in terms of DSC, ${{\text{HD}}}_{95}$, PDV, and MDA, respectively, with ${{\text{M}}}_{{\text{nnU}}-{\text{Net}}}$ showing underperformance. On the other hand, ${{\text{M}}}_{{\text{paU}}-{\text{Net}}}$ showed slightly improved performance than ${{\text{M}}}_{{\text{nnU}}-{\text{Net}}}$ and ${{\text{M}}}_{{\text{Seg}}+{\text{Spleen}}}$ in case of segment 1 and 2. Specifically, mean DSC of segment 1 from ${{\text{M}}}_{{\text{paU}}-{\text{Net}}}$ was greater than that of ${{\text{M}}}_{{\text{Seg}}+{\text{Spleen}}}$ by 8%. Similarly, mean DSC of segment 2 from ${{\text{M}}}_{{\text{paU}}-{\text{Net}}}$ was greater than that of ${{\text{M}}}_{{\text{Seg}}+{\text{Spleen}}}$ by 5%. However, such magnitude of discrimination was not observed in segment 1 and 4 in terms of ${{\text{HD}}}_{95}$. ${{\text{M}}}_{{\text{paU}}-{\text{Net}}}$ showed mean ${{\text{HD}}}_{95}$ greater than that of other two models by 10 mm and 6 mm in case of segments 1 and 4, respectively. Likewise, the mean PDV were larger than two models by 9% for segment 4. Additionally, ${{\text{M}}}_{{\text{paU}}-{\text{Net}}}$ showed MDA was within 1 mm for all segments when compared with the ${{\text{M}}}_{{\text{Seg}}+{\text{Spleen}}}$.

No ${{\text{M}}}_{{\text{Vess}}}$ model was trained, and no qualitative evaluation was performed because there is no vessel information on the non-CECT images.

#### Intra- and inter-observer analysis

The intraobserver mean DSCs on contours drawn by radiologist MA were of 0.88, 0.88, 0.94, 0.92, and 0.99 in segments 1, 2, 3, 4, 5–8, respectively. Likewise, the interobserver mean DSCs in between the contours drawn by radiologists JAMS and MA were 0.82, 0.85, 0.91, 0.88, and 0.96, respectively.

## Discussion

In this study, we have developed a clinically translatable model that can be used to auto-contour the liver segments and spleen on abdominal CT images. We validated all models on a validation set (${{\text{C}}}_{{\text{RTVal}}}$) of 40 CECT of patients with primary and metastatic liver tumors to identify the best model. We also assessed all models on various test sets (N = 78) shown in Fig. 1. First, we demonstrated that 3D full resolution architecture of nnU-Net outperformed 3D attention U-Net (paU-Net) by 2–5% in DSC across all liver segments. We also investigated the impact of adding segmentation of the vessels and spleen to aid in segmenting the liver segments and observed no major performance change between the models. Our final model can segment liver segments 1, 2 ,3 ,4 and 5–8 and the spleen with an average mean DSC of 0.89 and 0.99 across liver segments and spleen, respectively. We demonstrated that our model can be used in the clinical environment for surgical planning (mean DSC = 0.91) and for PVE patients (overall Likert score $\ge 4$ for 95%). To our knowledge, this is the first study to develop a single model to contour liver segments and spleen which is validated across primary/secondary liver cancers patients and across both contrast and non-contrast images.

Our final model is applicable in four clinical scenarios. First, the model can be used to auto-contour the segments of liver surgery patients where it can aid in estimating the volumetric change due to PVE and in overall resection planning, demonstrating an accuracy of 5.6% in overall median volume. Second, the model can be used to auto-contour liver segments in patients undergoing RT for liver cancer as studies^5,27,28^ have highlighted the importance of understanding liver segment regeneration for the optimization of RT plans. Third, the volume estimation from the model can be used in the prediction of cirrhosis and fibrosis as studies have reported that segment-volume ratio are significant predictors of cirrhosis/fibrosis^18,29^. Last, for the pathologies leading to hepatosplenomegaly, our model can be used to segment liver and spleen with higher accuracies in the case where segment 2 and 3 is abutted with spleen. Once our model is fully translated in the clinic, the utilization of model will allow improve efficiency, as the model can generate all its structure in 30–75 s per patient. The required time is very efficient compared to 90 min required in manual segmentation at our clinic and up to three minutes required in some of the semi-automatic segmentation methods^30^.

With regard to technical results, our first major observation was in the comparison of STAPLE vs. majority vote where we hypothesized that STAPLE > majority vote. This was confirmed based upon visual assessment that all 40 images in test set from ${{\text{C}}}_{{\text{RT}}}$ has at least one slice with increased zero valued pixel at the segment demarcation than STAPLE. The observation was expected because STAPLE assigns the label based on the probability values compared to a majority voting in SimpleITK (used in our study), which utilizes frequency of label which could lead to large number of undecided pixels. Second, in our architecture selection study, we observed that the nnU-Net architecture was superior with the paU-Net architecture demonstrating over segmentation of segment 1 including volume of segments 4, 5–8, and inferior venacava, and under segmentation in segment 3 with volume classified as segments 2 and 4. This could be due to less options in data augmentation in paU-Net than nnU-Net which greatly impacted the performance in the cases where vessel defining the segment boundaries deviated from the majority of the training data. Lastly, the paU-Net often failed to accurately contour segment 4, typically failing at the interface of the portal vein. The nnU-Net did not suffer from this uncertainty and therefore the accuracy improvement for segment 4 was the most significant, compared to the paU-Net model.

With regard to improvement in uncertainty, the statistical test showed no differences in models when spleen were added to the best architecture model. Specifically, in ${{\text{C}}}_{{\text{RT}}}$, for ${{\text{M}}}_{{\text{vess}}}\mathrm{ vs }{{\text{M}}}_{{\text{nnU}}-{\text{Net}}}$, 91% of the cases showed DSC differences within [-0.025,0.025]. The cases where DSC differences were larger (${{\text{M}}}_{{\text{nnU}}-{\text{Net}}}$>${{\text{M}}}_{{\text{vess}}}$) corresponded to errors in ${{\text{M}}}_{{\text{vess}}}$ due to over segmentation of segment 3 to segment 2 in two cases, over segmentation and under segmentation of segments 5–8 over 4 in one case. Similar trends were observed for ${{\text{M}}}_{{\text{vess}}}\mathrm{ vs }{{\text{M}}}_{{\text{seg}}+{\text{spleen}}}$, to suggest a preference for ${{\text{M}}}_{{\text{seg}}+{\text{spleen}}}$. However, most of the contrast in performance was observed in segment 1, 2, and 4 (Table S5). In ${{\text{M}}}_{{\text{seg}}+{\text{spleen}}}$ vs. ${{\text{M}}}_{{\text{nnU}}-{\text{Net}}}$, we argued ${{\text{M}}}_{{\text{seg}}+{\text{spleen}}}$ was similar to ${{\text{M}}}_{{\text{nnU}}-{\text{Net}}}$ (Table 7 and Table S4), however, we selected ${{\text{M}}}_{{\text{seg}}+{\text{spleen}}}$ because of slightly improved performance. Quantitatively, we observed in Table 7 that descriptive statistics of the results were similar except segment 5–8 of ${{\text{M}}}_{{\text{seg}}+{\text{spleen}}}$ where minimum DSC and maximum ${{\text{HD}}}_{95}$ improved by 0.05 and ~ 9 mm upon addition of spleen. Upon qualitative assessment of those cases, the improvement in ${{\text{M}}}_{{\text{seg}}+{\text{spleen}}}$ was due to lesser under segmentation of segment 5–8 compared to ${{\text{M}}}_{{\text{nnU}}-{\text{Net}}}$. Next, our validation set included N = 8/40 cases of segment 2 and 3 hypertrophy. In N = 7/8, there was no difference in segment 2 and 3 i.e., both models showed reasonable segmentation without any over or under segmentations. In N = 1/7, ${{\text{M}}}_{{\text{nnU}}-{\text{Net}}}$ showed under segmentation of segment 2 next to spleen but segmentations from ${{\text{M}}}_{{\text{seg}}+{\text{spleen}}}$ were improved on the same slices. Although our hypothesis that including the spleen in the model would be better than one without spleen was not supported because both models showed reasonable performance on cases with segment 2/3 hypertrophy we still selected model with spleen as our final model as this model has wider application and can be also used to estimate the severity of cirrhosis/fibrosis if needed in the patient undergoing liver surgery or RT.

${{\text{M}}}_{{\text{seg}}+\mathrm{spleen }},$ the final model showed excellent performance on the ${{\text{C}}}_{{\text{LS}}}$ patients for all segments except segment 2 where mean DSC was 0.85 and mean ${{\text{HD}}}_{95}$ was 9.4 mm. Furthermore, the subjective analysis showed that except segment 2/3, more than 70% of all cases received overall score $\ge 4$ on Likert score. This is likely due to the uncertainties in the boundary of segment 2/3. While the uncertainties are primarily attributed to performance of the model, it is also important to note that the opacification of the veins plays a great role in the ability of radiologist to evaluate the segmentation. The radiologist (SY) reported that N = 16/33 images were arterial phase images leading to a reduced confidence level in the evaluation of the contours as the portal venous branches are not well opacified and localized on the arterial phase images. Additionally, the visual assessment also showed that 5/33 of ${{\text{C}}}_{{\text{LS}}}$ showed holes or under segmentation in segment 5–8 due to photon starvation from metal artifact of the embolization coil/stent (N = 4/5) and tumor hole (N = 1/5).Furthermore, another N = 2/33 cases showed holes and under segmentation in segment 4 due to photon starvation from metal artifacts. Lastly, we observed slightly lower DSC in CC compared to CRM primarily because of portal hypertension in CC which could lead to enlarged spleen and could affect the contour performance. This was supported when obtained a difference of 13 cc between mean volume of both cancer types. Comparatively, since our final model showed better DSC in the case of CC opposed to HCC by approximately 2%, it could be argued that the severity of underlying disease which affects liver texture on CT across different cancer types could also impact vessels and hence the contours. Therefore, one would expect a DSC performance trend of colorectal metastasis patients > Cholangiocarcinoma > Hepatocellular carcinoma. However, since the number of patients in Cholangiocarcinoma in ${{\text{C}}}_{{\text{LS}}}$ is smaller (5 vs. 22), we cannot state a robust conclusion. In comparison with other models, our best model outperformed paU-Net and vessel-based model mostly on segment 1, 4 and 2, 3, 4, respectively but not on segment 5–8. This could be because segment 5–8 is the largest structure which means it is less sensitive to change in the vessel structures and includes more features. This requires lesser optimization in the model which means model less robust models such ${{\text{M}}}_{{\text{paU}}-{\text{Net}}}$ could also show better performance.

Next, in ${{\text{C}}}_{{\text{CH}}},$ ${{\text{M}}}_{{\text{Seg}}+{\text{Spleen}}}$ showed overall mean DSC of 0.87 which was smaller than the observed results on the ${{\text{C}}}_{{\text{LS}}}$ and ${{\text{C}}}_{{\text{RT}}}{\text{Val}}$ sets. Specifically, poor results were confined to segment 1 thru 4. The reason behind such observation was uncertainties in the boundaries of the segments in most of the cases. The images in challenge dataset also include cases with large and multiple tumors in the which could potentially lead to vessel occlusion and/or unremarkable opacification of the vessels on CT scans. Further, upon visual assessment, N = 4/25 cases of ${{\text{C}}}_{{\text{CH}}}$ showed under and over segmentation. Specifically, N = 3/4 showed under segmentation in segment 2, 4, and 5–8 dues to tumor and diseases, and N = 1/4 segment showed over segmentation to heart.

In ${{\text{C}}}_{{\text{PVE}}}$, we observed that the overall mean DSC of segments was 0.87 which is primarily because of poor performance in the segment 2. Upon visual assessment, we found N = 20/20 images showed inconsistency between the segment 2–3 boundary of ground-truth and prediction. The boundary of segments 2 and 3 is dictated by the portal veins in the left liver, and the architecture of those veins exhibit higher variation across patient population due to disease in liver. Another reason is segmental hypertrophy which could result in under and over segmentation of a specific segments. The volume of segment 2 from our best model in ${{\text{C}}}_{{\text{PVE}}}$ is 148 $\pm$ 73 cc and the ground-truth volume of normal liver from CHAOS dataset is 88 $\pm$ 36 cc which supports there is hypertrophy of segment 2. Next, regarding the effect of metallic artifacts, we found that N = 17/20 patients of ${{\text{C}}}_{{\text{PVE}}}$ had embolization coils spanning segment 5–8 and 4 with mostly localized in segment 5–8. N = 2/17 were immune from the impact of metal artifacts. However, in the remaining N = 15/17, both segments 4 (N = 3/15) and 5–8 (N = 15/15) showed holes in contours due to photon starvation arising from metal artifacts. This was expected because our training dataset did not include patients undergoing portal vein embolization. Lastly, the stratified DSC analysis for different cancer types showed the model performed better on CC (N = 3) patients than CRM (N = 17) patients by 2–4% which is not consistent with our observation in the ${{\text{C}}}_{{\text{RTVal}}}$.

Next, in the perturbation analysis, we observed that ${{\text{M}}}_{{\text{Seg}}+{\text{Spleen}}}$ was still better than the other two models in terms of DSC, HD_95_, and PDV across all segments except segment 1 and 2. In segment 1, and 2, ${{\text{M}}}_{{\text{paUNet}}}$ showed slightly better performance (p < 0.05). However, this was contradicted when we assessed the MDA which was higher for ${{\text{M}}}_{{\text{paUNet}}}$. Therefore, we attribute the observation of DSC for segment 1 mostly because of attention mechanism due to absence of contrast and randomness in the data. Overall, we argue that our best model could be potentially used on non-contrast images of same examination in clinic for segments 3, 4, 5–8 and spleen. For segments 1, and 2, minimum interventions from radiologist would be required to correct the contours. Lastly, since the non-contrast images are hardly used to discriminate tumor types, we did not perform stratified DSC analysis on non-contrast cases.

Comparing the performance of final model across validation and various test sets in Tables 6, 7, 8 and 9, we found that model performs best on ${{\text{C}}}_{{\text{LS}}}$ as evidenced by improved segment mean DSC (2–6%) than other cohorts. This could be attributed to the fact that surgery patients have less severe pathologies (e.g., surgery is typically a first-line therapy for smaller tumors) and minimal fewer artifacts than patients undergoing radiotherapy or portal vein embolization or patients in challenge cohorts. Further, we also observed that mean segmental DSC of ${{\text{C}}}_{{\text{RTVal}}}$ and ${{\text{C}}}_{{\text{LS}}}$ were slightly better than ${{\text{C}}}_{{\text{CH}}}$ and ${{\text{C}}}_{{\text{PVE}}}$. Specifically, while performance in segment 1 is within mean DSC of 2% across the datasets, segments 2, 3, and 4 showed lesser mean DSCs (up to 6%) which is attributed to fact that ${{\text{C}}}_{{\text{CH}}}$ dataset has larger and numerous tumors, larger slice thickness. For segment 5–8, ${{\text{C}}}_{{\text{PVE}}}$ showed lesser mean DSC by 3–5% due to presence of under segmentation or holes in segment 5–8 arising from metallic artifacts.

Considering the above analysis, our study has three limitations (1) segmentation of combined segments 5–8, (2) failure of the model on segments with metal artifacts, and (3) uncertainty in the segment 2 and 3 boundaries. For (1), clinical practice for surgical planning dictated our segmentation selection and the combination of segments 5–8. In addition, in our experience, there is substantial variability in the manual contouring of these segments individually. For (2), the issue can be addressed by manually editing the failed contours in the cases with severe photon starvation and increasing the number of such cases in our training datasets. For the last issue, we could implement post-processing methods to automatically optimize the boundary of segment 2 and 3. In our clinic, the segmental boundaries are separated based on the branching of portal veins and the regions above the left portal vein branch are segment 2 whereas regions below the left portal vein branches are segment 3^31^. We can use our in-house tool to generate liver vessels on CT scans in post-processing phase^23^ and also implement vessel enhancements and active contour methods, as reviewed by Ciecholewski et. al. 2021^32^, to further enhance the vessels at the periphery of segment 2 and 3. Despite the limitations, our model performs comparable or improved accuracy in comparison with studies as shown in Table 11. Tian et al. 2019 reported the mean values across all segments and our results are in close agreement with their result, In comparison with Lee et al. 2022, our model demonstrated superior results on ${{\text{C}}}_{{\text{LS}}}$ in all segments (except segment 2) by 3–30%. For segment 2, our model showed inferior results by up to 6% which is attributed to the variability in the boundaries of the segments 2 and sensitivity of our model to the vessel architecture. Another reason is the difference in the underlying pathology of the literature compared to our datasets. Lee et al. 2022^18^ assessed their model performance on the patients with hepatitis C and cirrhosis, however, our ${{\text{C}}}_{{\text{LS}}}$ is dominantly CRM and CC patients (see Table 1). The severity of cancer is also known to cause cavernous transformation of the vessels which also leads to uncertainties in the segment 2 contours.

**Table 11:** Comparison of ${{\text{M}}}_{{\text{seg}}+{\text{spleen}}}$ with studies that developed liver w/wo spleen segmentation.

	Ours				Lee et al. 2022^18^		Tian et al. 2019^17^
	Mean (${{\text{C}}}_{{\text{RT}}}$ test, N = 33)	Median (${{\text{C}}}_{{\text{RT}}}$ test, N = 33)	Mean (${{\text{C}}}_{{\text{LS}}}$, N = 33)	Median (${{\text{C}}}_{{\text{LS}}}$, N = 33)	Median (Data 1, N = 35)	Median (Data 1, N = 35)	Mean
Seg 1	0.89	0.93	0.912	0.94	0.64	0.66	0.9246
Seg 2		0.86		0.86	0.91	0.92	
Seg 3		0.91		0.91	0.88	0.88	
Seg 4		0.90		0.92	0.82	0.85	
Seg 5–8*		0.97		0.98	0.86	0.84	
Spleen	0.99	0.99	0.91	0.96	0.96	0.95	

*Lee et al. 2022 reported separate results of segment 5 thru 8. We averaged the reported median values.

## Conclusion

In this study, we developed and validated to a clinically acceptable accuracy, a fully automated model that can auto-contour liver segments and spleen on CECT images. We found that implementing the attention mechanism in 3D U-Net did not improve the performance when compared with the 3D full-resolution nnU-Net. We also identified that the addition of segmenting the vessels and spleen did not have large impact on accuracy of segment contours. The application of the model is primarily intended for use with patients undergoing assessment for liver surgery or liver radiotherapy, but the model can be used in any clinical scenario where there is a need for segment contouring on CECT. Upon assessing our model on patients undergoing portal-vein embolization, we conclude that contouring is significantly impacted by presence of metallic artifacts leading to holes in the contours. However, inclusion of such patients in the training may improve performance in the future. Lastly, with regard to non-contrast images, we conclude that our final model can contours segments with accuracies sufficient enough for clinical use with review and possibly moderate interventions from radiologist.

### Supplementary Information

Supplementary Information.

## Data Availability

The 3D-IRCADb-01 data can be accessed at https://www.ircad.fr/research/data-sets/liver-segmentation-3d-ircadb-01/. The 3D-IRCADb-02 dataset can be accessed at https://www.ircad.fr/research/data-sets/respiratory-cycle-3d-ircadb-02/. The task 8 Medical Imaging Decathlon Challenge dataset can be accessed at http://medicaldecathlon.com/dataaws/. The CHAOS dataset can be accessed at https://chaos.grand-challenge.org/Download/. The internal liver CT data used during our study are available upon reasonable request in compliance with instuitutional IRB requirements.
