# Supplementary material for: Fully automated deep learning based auto-contouring of liver segments and spleen on contrast-enhanced CT images
Source: Sci Rep. 2024 Feb 26;14:4678. doi: 10.1038/s41598-024-53997-y (PMC10967337; doi:10.1038/s41598-024-53997-y)
Supplement: Supplementary file 1 — Supplementary Information. [file 41598_2024_53997_MOESM1_ESM.docx]

**Supplementary Document**


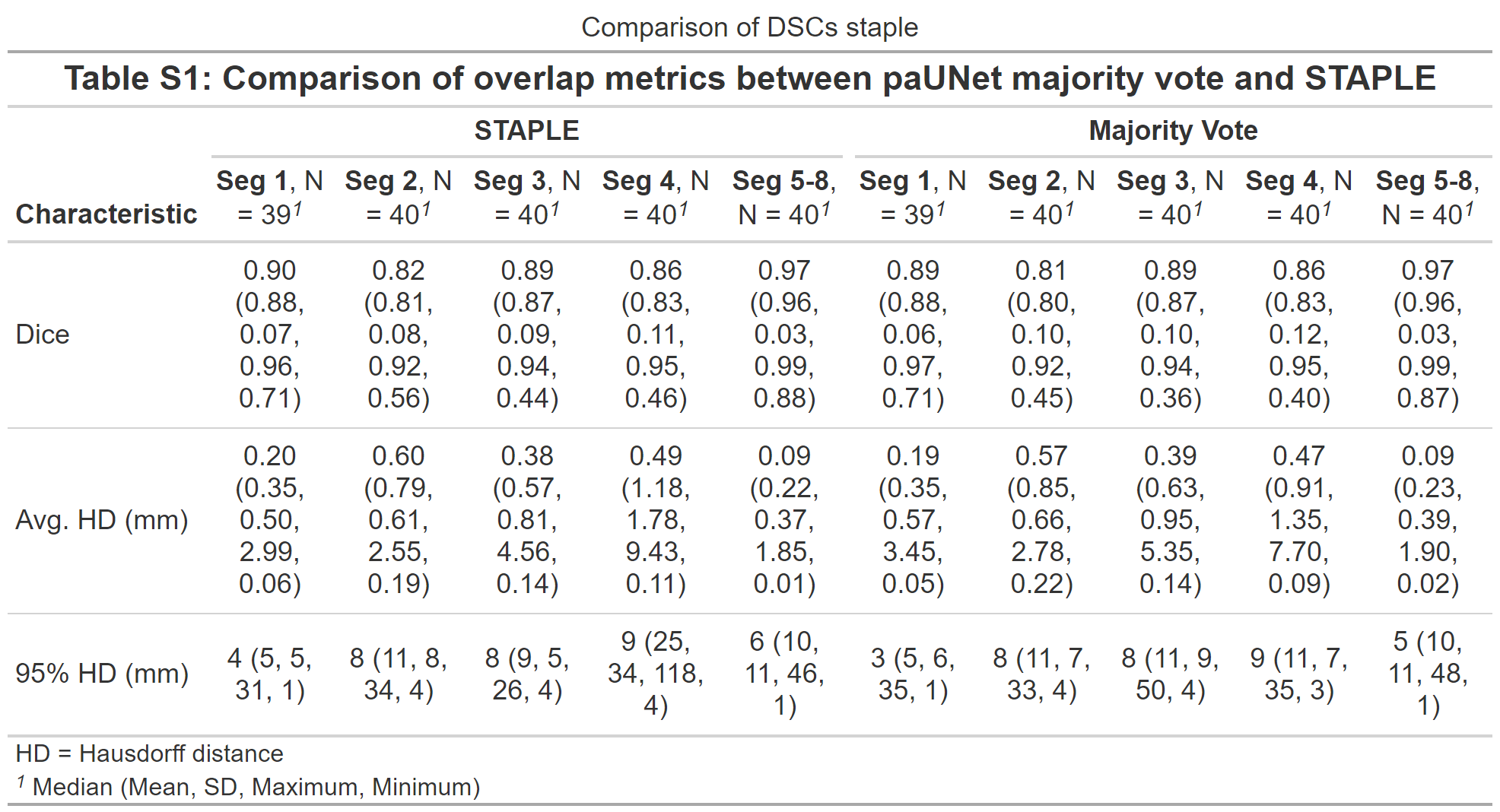


**
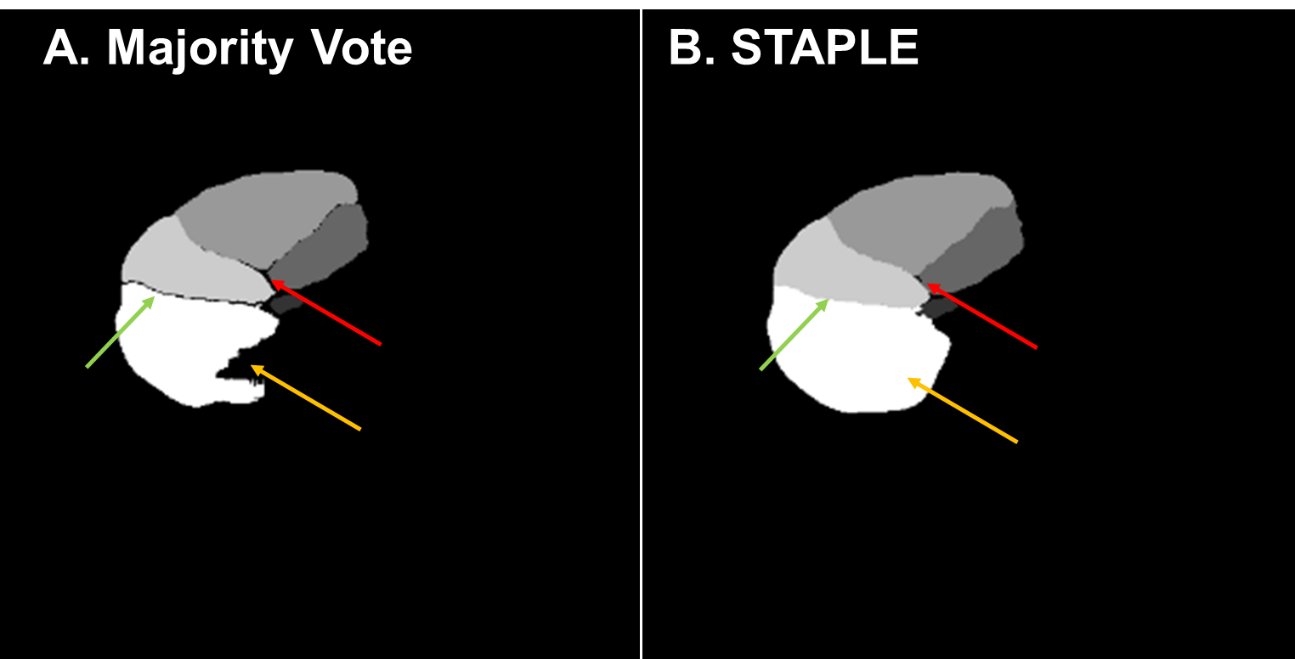
**

**Figure S1.** VV-slicer rendering of images showing comparison of segments boundaries from Majority Vote (A) and STAPLE (B) algorithm. Green, Red, and Orange arrow across two images highlights the decreased 0 pixels from STAPLE

| **Table S2: comparison of** $\mathbf{M}_{\mathbf{nnUNet}}$ **and** $\mathbf{M}_{\mathbf{paUNet}}$ **through frequency of cases meeting DSC bin criteria** | | | | | | | | | | |
| --- | --- | --- | --- | --- | --- | --- | --- | --- | --- | --- |
|  | **Seg 1** | | **Seg 2** | | **Seg 3** | | **Seg 4** | | **Seg 5-8** | |
|  | $\mathbf{M}_{\mathbf{nnUNet}}$ | $\mathbf{M}_{\mathbf{paUNet}}$ | $\mathbf{M}_{\mathbf{nnUNet}}$ | $\mathbf{M}_{\mathbf{paUNet}}$ | $\mathbf{M}_{\mathbf{nnUNet}}$ | $\mathbf{M}_{\mathbf{paUNet}}$ | $\mathbf{M}_{\mathbf{nnUNet}}$ | $\mathbf{M}_{\mathbf{paUNet}}$ | $\mathbf{M}_{\mathbf{nnUNet}}$ | $\mathbf{M}_{\mathbf{paUNet}}$ |
| [0.025,0.05) | **7** | 0 | 9 | 2 | 7 | 1 | 8 | 2 | 4 | 0 |
| [0.05,0.1) | 8 | 0 | 4 | 2 | 3 | 1 | 10 | 0 | 1 | 1 |
| >0.1 | 1 | 0 | 4 | 1 | 2 | 1 | 4 | 0 | 0 | 0 |
| Total | 16 | 0 | 17 | 5 | 12 | 3 | 22 | 2 | 5 | 1 |
| $f_{N_{nnUNet}:N_{paUNet}}$ | 16:0 | | 17:5 (3.4) | | 4:1 | | 11:1 | | 5:1 | |

| **Table S3: comparison of** $\mathbf{M}_{\boldsymbol{vess}}$ **and** $\mathbf{M}_{\mathbf{nnUNet}}$ **through frequency of cases meeting DSC bin criteria** | | | | | | | | | | |
| --- | --- | --- | --- | --- | --- | --- | --- | --- | --- | --- |
|  | **Seg 1** | | **Seg 2** | | **Seg 3** | | **Seg 4** | | **Seg 5-8** | |
|  | $\mathbf{M}_{\mathbf{vess}}$ | $\mathbf{M}_{\mathbf{nnUNet}}$ | $\mathbf{M}_{\mathbf{vess}}$ | $\mathbf{M}_{\mathbf{nnUNet}}$ | $\mathbf{M}_{\mathbf{vess}}$ | $\mathbf{M}_{\mathbf{nnUNet}}$ | $\mathbf{M}_{\mathbf{vess}}$ | $\mathbf{M}_{\mathbf{nnUNet}}$ | $\mathbf{M}_{\mathbf{vess}}$ | $\mathbf{M}_{\mathbf{nnUNet}}$ |
| [2.5,5) | 1 | 3 | 0 | 3 | 1 | 1 | 0 | 5 | 0 | 0 |
| [5,10) | 0 | 0 | 1 | 1 | 0 | 1 | 0 | 1 | 1 | 0 |
| >10 | 0 | 0 | 0 | 0 | 0 | 1 | 0 | 1 | 0 | 1 |
| Total | 1 | 3 | 1 | 4 | 1 | 3 | 0 | 7 | 1 | 1 |
| $f_{M_{vess}:M_{nnU-Net}}$ | 1:3 (0.33) | | 1.4 (0.25) | | 1:3 (0.33) | | 0:7 | | 1:1 | |

| **Table S4: comparison of** $\mathbf{M}_{\boldsymbol{Seg+Spleen}}$ **and** $\mathbf{M}_{\mathbf{nnUNet}}$ **through frequency of cases meeting DSC bin criteria** | | | | | | | | | | |
| --- | --- | --- | --- | --- | --- | --- | --- | --- | --- | --- |
|  | **Seg 1** | | **Seg 2** | | **Seg 3** | | **Seg 4** | | **Seg 5-8** | |
|  | $\mathbf{M}_{\mathbf{seg+spleen}}$ | $\mathbf{M}_{\mathbf{nnUNet}}$ | $\mathbf{M}_{\mathbf{seg+spleen}}$ | $\mathbf{M}_{\mathbf{nnUNet}}$ | $\mathbf{M}_{\mathbf{seg+spleen}}$ | $\mathbf{M}_{\mathbf{nnUNet}}$ | $\mathbf{M}_{\mathbf{seg+spleen}}$ | $\mathbf{M}_{\mathbf{nnUNet}}$ | $\mathbf{M}_{\mathbf{seg+spleen}}$ | $\mathbf{M}_{\mathbf{nnUNet}}$ |
| [0.025,0.05) | 1 | **1** | 1 | 1 | 0 | 0 | 1 | 0 | 0 | 0 |
| [0.05,0.1) | 0 | 0 | 0 | 0 | 0 | 0 | 0 | 0 | 1 | 0 |
| >0.1 | 0 | 0 | 0 | 0 | 0 | 0 | 0 | 0 | 0 | 0 |
| Total | 1 | 1 | 1 | 1 | 0 | 0 | 1 | 0 | 1 | 0 |
| $f_{M_{seg+spleen}:M_{nnU-Nettt}}$ | 1:1 | | 1:1 | | 0:0 | | 1:0 | | 1:0 | |

| **Table S5: comparison of** $\mathbf{M}_{\boldsymbol{Seg+Spleen}}$ **and** $\mathbf{M}_{\boldsymbol{vess}}$ **through frequency of cases meeting DSC bin criteria** | | | | | | | | | | |
| --- | --- | --- | --- | --- | --- | --- | --- | --- | --- | --- |
|  | **Seg 1** | | **Seg 2** | | **Seg 3** | | **Seg 4** | | **Seg 5-8** | |
|  | $\mathbf{M}_{\mathbf{seg+spleen}}$ | $\mathbf{M}_{\mathbf{vess}}$ | $\mathbf{M}_{\mathbf{seg+spleen}}$ | $\mathbf{M}_{\mathbf{vess}}$ | $\mathbf{M}_{\mathbf{seg+spleen}}$ | $\mathbf{M}_{\mathbf{vess}}$ | $\mathbf{M}_{\mathbf{seg+spleen}}$ | $\mathbf{M}_{\mathbf{vess}}$ | $\mathbf{M}_{\mathbf{seg+spleen}}$ | $\mathbf{M}_{\mathbf{vess}}$ |
| [2.5,5) | 3 | **0** | 4 | 2 | 1 | 1 | 4 | 1 | 0 | 0 |
| [5,10) | 0 | 0 | 1 | 0 | 1 | 0 | 2 | 0 | 0 | 0 |
| >10 | 0 | 0 | 0 | 0 | 0 | 1 | 0 | 1 | 0 | 1 |
| Total lead | 3 | 0 | 5 | 2 | 2 | 2 | 6 | 2 | 0 | 1 |
| $f_{M_{seg+spleen}:M_{\mathrm{vess}}}$ | 3:0 | | 5:2 (2.5) | | 1:1 (1.0) | | 3:1 (3.0) | | 0:1 | |
